# HDR, the last enzyme in the MEP pathway, differently regulates isoprenoid biosynthesis in two woody plants

**DOI:** 10.1093/plphys/kiad110

**Published:** 2023-02-22

**Authors:** Toni Krause, Piera Wiesinger, Diego González-Cabanelas, Nathalie Lackus, Tobias G Köllner, Thomas Klüpfel, Jonathan Williams, Johann Rohwer, Jonathan Gershenzon, Axel Schmidt

**Affiliations:** Department of Biochemistry, Max Planck Institute for Chemical Ecology, Hans-Knöll-Str. 8, 07745 Jena, Germany; Department of Biochemistry, Max Planck Institute for Chemical Ecology, Hans-Knöll-Str. 8, 07745 Jena, Germany; Department of Biochemistry, Max Planck Institute for Chemical Ecology, Hans-Knöll-Str. 8, 07745 Jena, Germany; Department of Biochemistry, Max Planck Institute for Chemical Ecology, Hans-Knöll-Str. 8, 07745 Jena, Germany; Department of Biochemistry, Max Planck Institute for Chemical Ecology, Hans-Knöll-Str. 8, 07745 Jena, Germany; Department of Atmospheric Chemistry, Max Planck Institute for Chemistry, Hahn-Meitner-Weg 1, Germany; Department of Atmospheric Chemistry, Max Planck Institute for Chemistry, Hahn-Meitner-Weg 1, Germany; Department of Biochemistry, Stellenbosch University, Private Bag X1, Matieland, 7602 Stellenbosch, South Africa; Department of Biochemistry, Max Planck Institute for Chemical Ecology, Hans-Knöll-Str. 8, 07745 Jena, Germany; Department of Biochemistry, Max Planck Institute for Chemical Ecology, Hans-Knöll-Str. 8, 07745 Jena, Germany

## Abstract

Dimethylallyl diphosphate (DMADP) and isopentenyl diphosphate (IDP) serves as the universal C_5_ precursors of isoprenoid biosynthesis in plants. These compounds are formed by the last step of the 2-*C*-methyl-D-erythritol 4-phosphate (MEP) pathway, catalyzed by (*E*)-4-hydroxy-3-methylbut-2-en-1-yl diphosphate reductase (HDR). In this study, we investigated the major HDR isoforms of two woody plant species, Norway spruce (*Picea abies*) and gray poplar (*Populus × canescens*), to determine how they regulate isoprenoid formation. Since each of these species has a distinct profile of isoprenoid compounds, they may require different proportions of DMADP and IDP with proportionally more IDP being needed to make larger isoprenoids. Norway spruce contained two major HDR isoforms differing in their occurrence and biochemical characteristics. *Pa*HDR1 produced relatively more IDP than *Pa*HDR2 and it encoding gene was expressed constitutively in leaves, likely serving to form substrate for production of carotenoids, chlorophylls, and other primary isoprenoids derived from a C_20_ precursor. On the other hand, Norway spruce *Pa*HDR2 produced relatively more DMADP than *Pa*HDR1 and its encoding gene was expressed in leaves, stems, and roots, both constitutively and after induction with the defense hormone methyl jasmonate. This second HDR enzyme likely forms a substrate for the specialized monoterpene (C_10_), sesquiterpene (C_15_), and diterpene (C_20_) metabolites of spruce oleoresin. Gray poplar contained only one dominant isoform (named *Pc*HDR2) that produced relatively more DMADP and the gene of which was expressed in all organs. In leaves, where the requirement for IDP is high to make the major carotenoid and chlorophyll isoprenoids derived from C_20_ precursors, excess DMADP may accumulate, which could explain the high rate of isoprene (C_5_) emission. Our results provide new insights into the biosynthesis of isoprenoids in woody plants under conditions of differentially regulated biosynthesis of the precursors IDP and DMADP.

## Teaser

Isoprenoid biosynthesis in woody plants with a distinct profile of isoprenoid compounds is regulated by the ratio of the precursors isopentenyl diphosphate and dimethylallyl diphosphate.

## Introduction

The isoprenoids, with more than 40,000 structures known, represent the largest group of plant metabolites. Also known as terpenes or terpenoids, plant isoprenoids include primary metabolites, such as chlorophylls, carotenoids, ubiquinones, cytokinins, gibberellins, brassinosteroids and abscisic acid, and an enormous variety of specialized metabolites (Pérez-Gil et al. 2017; Pichersky and Raguso 2018). Specialized isoprenoids often function in interactions with other organisms, including herbivores, pathogens, and other plants (Ashour et al. 2010).

Isoprenoids are formed from branched-chain C_5_ units and are classified by the number of these units they contain as hemi- (C_5_), mono- (C_10_), sesqui- (C_15_), di- (C_20_), sester- (C_25_), tri- (C_30_), tetra- (C_40_) or polyterpenes (C_n_). Biosynthesis proceeds from the fusion of the C_5_ intermediates, dimethylallyl diphosphate (DMADP), and its double-bond isomer isopentenyl diphosphate (IDP), with additional IDP units, added to make larger isoprenoids (Bohlmann and Keeling 2008). The larger the terpene, the more IDP is required relative to DMADP. Therefore, the relative availability of DMADP and IDP may have a large impact on the types of isoprenoids that can be formed.

In plants, two distinct pathways synthesize DMADP and IDP. First, the cytosolic mevalonate (MVA) pathway starting from acetyl-coenzyme A generates IDP, which is further isomerized by isopentenyl diphosphate isomerase (IDI, EC: 5.3.3.2) to DMADP (Phillips et al. 2008). In plastids, the methylerythritol 4-phosphate (MEP) pathway synthesizes a mixture of DMADP and IDP with the product ratio altered by IDI-catalyzed isomerization as well (Pulido et al. 2012). Although the two pathways are spatially separated, exchange between the two pathways has been documented in a number of cases (Bick and Lange 2003; González-Cabanelas et al. 2015; Henry et al. 2018).

**Figure 1.**
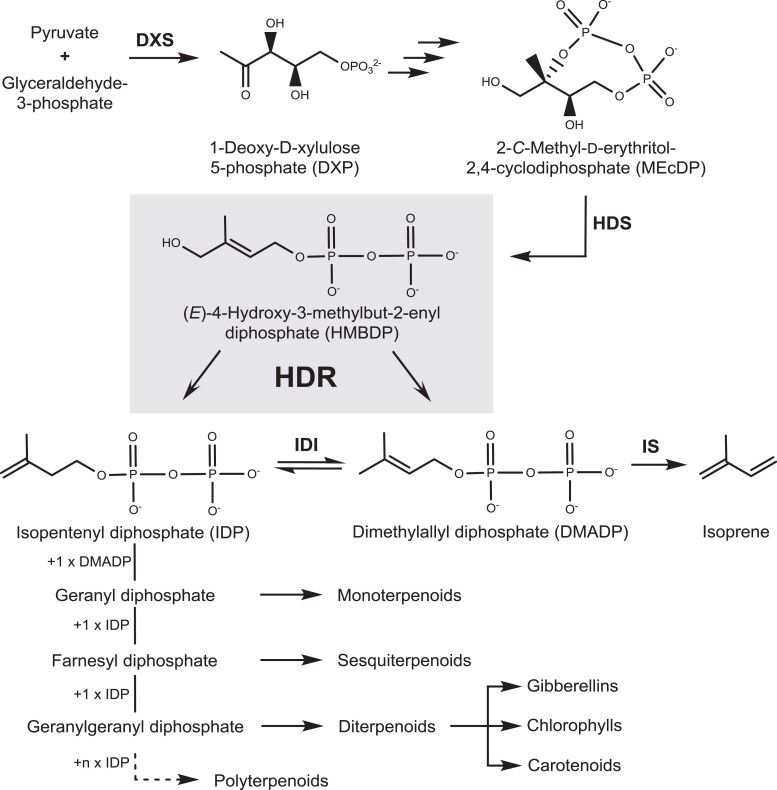
(*E*)-4-Hydroxy-3-methylbut-2-en-1-yl diphosphate reductase (HDR) functions as a central enzyme in isoprenoid biosynthesis in plants. HDR Is the terminal enzyme of the methylerythritol 4-phosphate (MEP) pathway, catalyzing the reaction of (*E*)-4-hydroxy-3-methylbut-2-enyl diphosphate (HMBDP) to dimethylallyl diphosphate (DMADP) and isopentenyl diphosphate (IDP). IDP can also be isomerized to DMADP by isopentenyl diphosphate isomerase (IDI). DMADP can be converted to isoprene under catalysis of isoprene synthase (IS). Biosynthesis of larger isoprenoids involves the formation of geranyl-, farnesyl- and geranylgeranyl diphosphate by catalyzing the condensation reaction of one, two or three IDP building blocks to DMADP, respectively. Prenyl diphosphates can be further metabolized to different classes of terpenes including hemi-, mono-, sesqui-, diterpenes, and larger polyterpenes. DXS: 1-deoxy-D-xylulose-5-phosphate synthase; HDS: (*E*)-4-hydroxy-3-methylbut-2-en-1-yl diphosphate synthase.

For the production of DMADP and IDP, the MEP pathway utilizes glyceraldehyde-3-phosphate and pyruvate as its initial substrates, converting them to 1-deoxy-D-xylulose-5-phosphate (DXP) catalyzed by DXP synthase (DXS, EC: 2.2.1.7). This first reaction is considered the rate-limiting step of the pathway, and its activity is regulated at the transcript and protein levels (Banerjee et al. 2013; Hemmerlin 2013; Wright et al. 2014). DXP is further processed to 2-*C*-methyl-D-erythritol-2,4-cyclodiphosphate (MEcDP) via four additional steps (Bitok and Meyers 2012). Then MEcDP is converted by *(E)*-4-hydroxy-3-methylbut-2-enyl diphosphate (HMBDP) synthase (HDS, EC: 1.17.7.1) to HMBDP, and finally, HMBDP reductase (HDR, EC: 1.17.7.4) converts HMBDP to a mixture of DMADP and IDP. The amounts and proportions of DMADP and IDP in plants are influenced by HDR catalysis, but also by IDI and the enzymes that form larger prenyl diphosphate intermediates. In addition, isoprene synthase (IS, EC: 4.2.3.27) converts DMADP directly to isoprene, a volatile C_5_ metabolite released by about 20% of all plant species in the world (Loreto and Fineschi 2015). A similar process that directly hydrolyzes IDP to a volatile, dephosphorylated product has not been discovered so far (Schnitzler et al. 2004; Vickers and Sabri 2015; Sharkey and Monson 2017) (Fig. 1).

In the last two decades, HDR has received attention as an enzyme for manipulating DMADP and IDP content and increasing MEP pathway flux in the biotechnological production of isoprenoids (Adam et al. 2002). It was found that bacterial HDR enzymes (designated “IspH”) yield DMADP:IDP ratios of approximately 1:5 as described for *Escherichia coli*, *Aquifex aeolicus* and *Plasmodium falciparum* (Adam et al. 2002; Altincicek et al. 2002; Wolff et al. 2003; Graewert et al. 2004; Roehrich et al. 2005). These studies of bacterial HDRs have provided useful insights into protein structure and reaction mechanisms. Yet, almost nothing is known about their plant homologs (Rohdich et al. 2003). Plant *HDR* genes showed the capability to rescue lethal *IspH* knock-out mutants of *E. coli* by complementation. This was described for genes from Arabidopsis (*Arabidopsis thaliana*) (Hsieh and Goodman 2005), stevia (*Stevia rebaudiana*) (Kumar and Kumar 2013), thunder god vine (*Tripterygium wilfordii*) (Cheng et al. 2017), gingko (*Ginkgo biloba*) (Kim et al. 2008), Japanese red pine (*Pinus densiflora*) (Kim et al. 2009), melon (*Cucumis melo* L.) (Saladié et al. 2014), taxus (*Taxus media*) (Sun et al. 2009) and various other species including sitka spruce (*Picea si tchensis*) (Bongers et al. 2020). A more in-depth characterization of a plant HDR was performed for *Ginkgo biloba*, which showed a DMADP:IDP ratio of 1:16 (Shin et al. 2017). However, in vitro characterization of this enzyme is challenging since enzymatic preparation has to be carried out under strict anaerobic conditions to avoid the oxidation of the [4Fe-4S] iron-sulfur cluster. This prosthetic group is essential for substrate binding and electron transfer in the reaction cycle, and rapidly decomposes upon contact with oxygen (Wolff et al. 2003; Graewert et al. 2009). In addition, accurate separation and quantification of the HDR products DMADP and IDP requires specialized chromatographic procedures (Krause et al. 2020). The levels of these products have sometimes been measured by various derivatization methods, which reduces accuracy and limits comparison within the literature.

Since different sizes isoprenoids require different proportions of DMADP:IDP in their formation, the product profile of HDR can influence the course of isoprenoid biosynthesis. Plants could conceivably use multiple HDR isoforms with different properties to optimize the formation of particular isoprenoid products. To date, all plants studied possess one or two genes encoding HDR. For example, *A. thaliana* has only a single copy of *HDR* and accumulates or emits only low amounts of monoterpene, sesquiterpene, and diterpene natural products (Chen et al. 2003; Vaughan et al. 2013; Wang et al. 2016). On the other hand, sweet wormwood (*Artemisia annua*), with two *HDR* genes biosynthesizes large amounts of isoprenoid natural products, including the well-known antimalarial drug artemisinin (Ma et al. 2015). Both *Ginkgo biloba* (Carrier et al. 1998) and *Cucumis melo* (Portnoy et al. 2008) also harbor two *HDR* genes and each produces sesquiterpene or diterpene natural products. For *G. biloba*, tissue-specific expression patterns have been described for both genes (Kim et al. 2008; Lu et al. 2008). Despite these reports, very little is known about how plant isoprenoid biosynthesis might be affected by the occurrence of multiple HDR isoforms. Silencing of *HDR* expression in plant species containing only a single *HDR* copy, such as *A. thaliana* and *Nicotiana benthamiana* (Page et al. 2004; Hsieh and Goodman 2005) resulted in growth deficiency and impaired chloroplast development caused by a lack of chlorophylls and carotenoids. In contrast, knock-down of an *HDR* in *A. annua*, a species with two *HDR* genes, showed only a minor reduction of isoprenoids, indicating compensation by the second gene (Ma et al. 2017).

In this work, we investigated the HDR complement of two woody plant species with different profiles of isoprenoid natural products. Norway spruce (*Picea abies*) synthesizes high amounts of an isoprenoid oleoresin composed of mono-, sesqui- and diterpenes (C_10_, C_15,_ and C_20_) (Martin et al. 2002; Keeling and Bohlmann 2006) and emits trace amounts of isoprene (C_5_) (Perreca et al. 2020). Gray poplar (*Populus* × *canescens*), a naturally occurring hybrid of aspen (*Populus tremula*) and white poplar (*Populus alba*), does not produce large amounts of isoprenoid natural products, but emits isoprene (C_5_) at a high rate and also low amounts of some monoterpene (C_10_) and sesquiterpene (C_15_) volatiles (Kesselmeier and Staudt 1999; McCormick et al. 2014). In addition, both *Picea abies* and *Populus* × *canescens*, like all other green plants, produce primary isoprenoids of which the major compounds are carotenoids and the side chain of the chlorophylls, both made from GGDP (C_20_). The differences in the isoprenoid composition of these species result in different demands for DMADP and IDP, and thus one might expect differences in the types of HDR present.

We studied the *HDRs* of *Picea abies* and *Populus* × *canescens* by cloning the two genes from each species and heterologously expressing the encoded proteins in *E. coli*. After extraction and purification under anaerobic conditions, the kinetic properties of each enzyme were determined in vitro. Then we knocked-down and over-expressed most of these genes in their hosts and looked for changes in the levels of the HDR substrates and products, other MEP pathway intermediates, and isoprenoid end products.

## Results

### Identification of *HDR* genes and their expression patterns

Using available *HDR* sequences from species of *Populus* and *Picea*, we obtained two *HDR* sequences each from *Populus* × *canescens* (gray poplar) and *Picea abies* (Norway spruce), and verified them by cloning and sequencing. The *HDR* sequences of *Populus* × *canescens* were identical at the amino acid level to those of *Populus trichocarpa*, and *HDR1* of *Picea abies* was identical to that of *Picea glauca*. Sequence alignment revealed that all of these HDR proteins contained a number of common features: putative transit peptide for plastidal localization; four equivalent cysteine residues, which are critical for catalytic activity as a part of the iron-sulfur cluster in the catalytic pocket; a conserved N-terminus found exclusively in HDRs of organisms carrying out oxygenic photosynthesis; and a few amino acids near the substrate binding site that are highly conserved in all plant HDRs as well as in bacterial IspHs (Graewert et al. 2004; Hsieh and Hsieh 2015; Cheng et al. 2017; Ma et al. 2017) (Supplemental Fig. S1). Phylogenetic analysis revealed that the sequences of these HDR isoforms differ in a species-dependent manner. While the two HDRs of *Picea abies* show a sequence similarity of only 66%, the sequences of *Populus* × *canescens* share nearly 88% identity. When these sequences were included in a phylogenetic tree with other plant HDRs, gymnosperms can be seen to have evolved HDR putative paralogs early in evolution, which then further differentiated. In contrast, angiosperms show a more recent origin of the HDR homologs, which may have led to their substantially higher sequence similarity (Saladié et al. 2014) (Supplemental Fig. S2, A and B).

**Figure 2.**
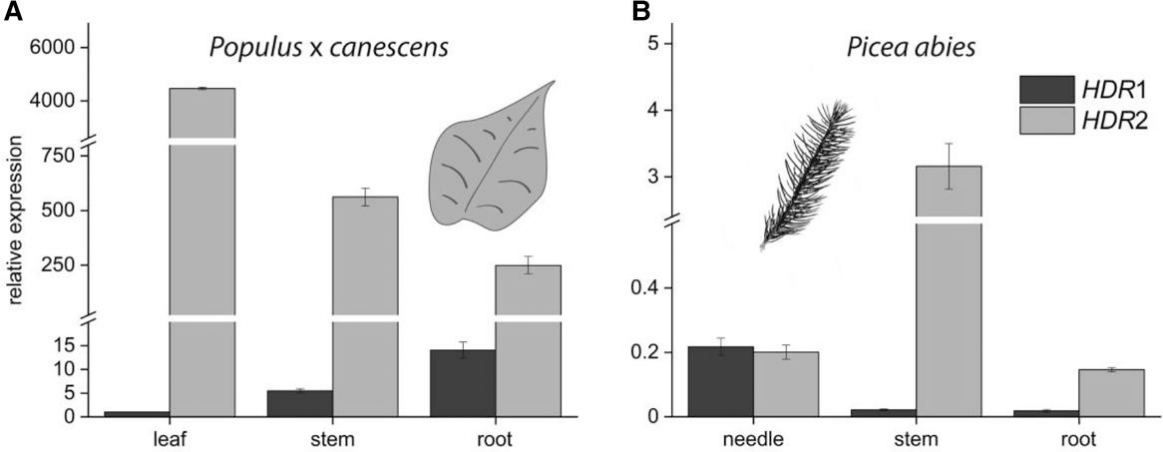
RT-qPCR analysis of *HDR*1 and *HDR*2 expression in different organs of *Populus* × *canescens* and *Picea abies. PcHDR*2 expression in *Populus* × *canescens* is many-fold greater than that of *PcHDR*1 in all organs tested, making it virtually the only active isoform in the plant **A)**. In *Picea abies*, *PaHDR*2 Is mainly expressed in spruce stems and roots, while *PaHDR*1 shows highest expression in needles **B)**. These differences in *HDR* isoform expression point towards species-specific regulation of the production of DMADP and IDP. Values are given as means ± standard deviation of three biological replicates, measured as technical triplicates. Statistical analysis was performed by using Student's t-test, letters represent statistical differences between the tissues; *P* ≤ 0.05.

To study the transcript abundance of the *HDR* genes in poplar, RT-qPCR analysis in *Populus* × *canescens* and RNA sequencing (RNA-Seq) analysis in *Populus trichocarpa* were performed. *Populus* × *canescens* displayed large differences in transcript levels between organs and between isoforms. *PcHDR*2 was expressed in leaf, stem, and root tissues at a level more than 100-fold greater than *PcHDR*1 (Fig. 2A). In *Populus trichocarpa*, *PtHDR*1 was also expressed at lower levels than *PtHDR*2, but in the roots these differences were much less than those in *Populus* × *canescens* roots (Supplemental Fig. S3, A to D).

To check the inducibility of gene expression by biotic and abiotic stresses, the levels of *PtHDR*1 and *PtHDR*2 transcripts in *Populus trichocarpa* leaves and roots were measured after leaf herbivory by *Lymantria dispar* (spongy moth) caterpillars, treatment of shoot and root with jasmonic acid, and root infection by the oomycete *Phytophthora cactorum*. After caterpillar feeding, *PtHDR*2 expression increased by a factor of 2.4 in leaves, whereas *PtHDR*1 expression levels were not significantly changed. (Supplemental Fig. S3A). After jasmonic acid treatment, both *PtHDR*1 and *PtHDR*2 expression increased by a factor of approximately 2 in leaves (Supplemental Fig. S3B). In roots, jasmonic acid treatment increased *PtHDR*1 expression by a factor of nearly 2, whereas *PtHDR*2 increased by a factor of over 4 (Supplemental Fig. S3D). After oomycete infestation of roots, both *PtHDR*1 and *PtHDR*2 exhibited higher gene expression levels in the roots, but these were only significant for the latter (Supplemental Fig. S3C).

In *Picea abies, PaHDR*2 gene expression was dominant to *PaHDR*1 in stems and roots, but the genes showed equal expression in needles (Fig. 2B). Plants treated with the defense hormone analog methyl jasmonate (MJ) showed induction of *PaHDR*2 gene expression in all tested tissues, peaking two days after application and decreasing towards constitutive levels after six days. *PaHDR*1 transcript levels were induced by MJ exclusively in roots, implying a regulatory function for HDR in this organ (Supplemental Fig. S3E).

### Biochemical characterization of HDR recombinant proteins

**Figure 3.**
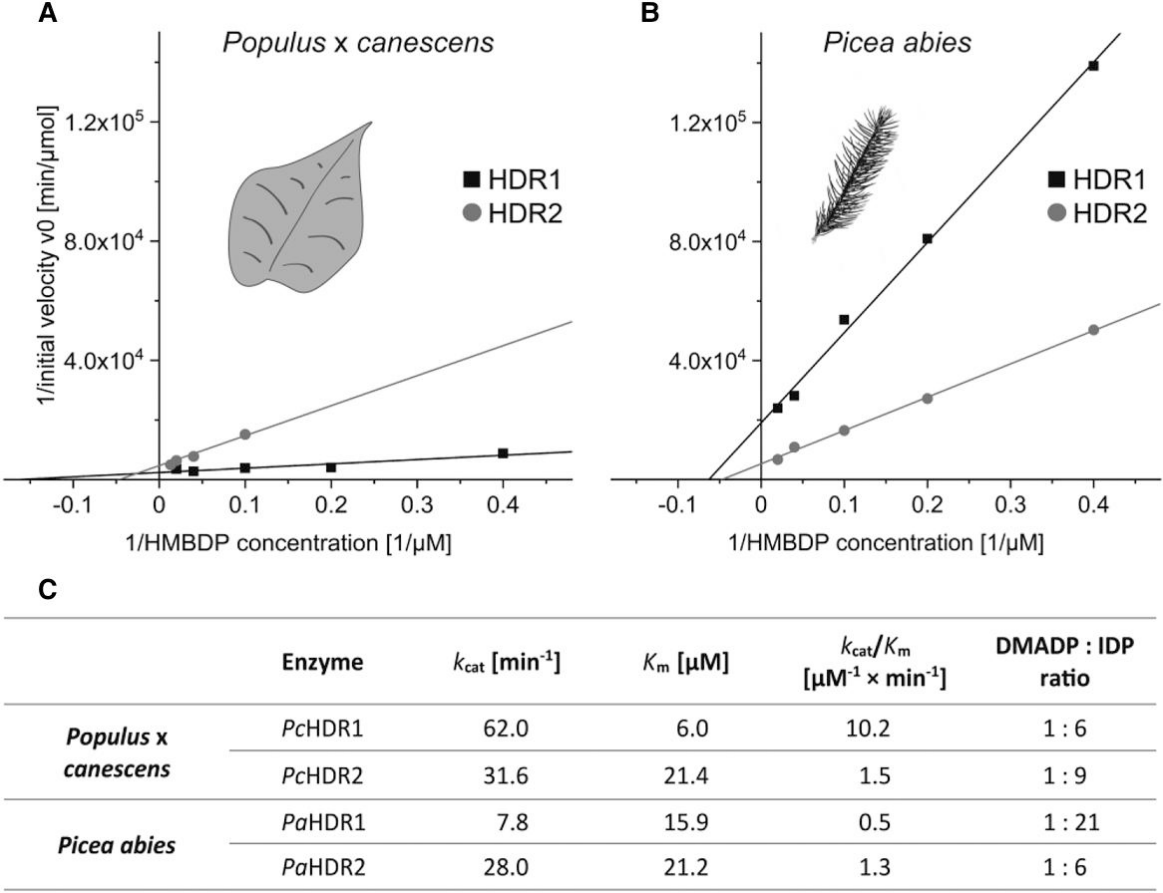
Kinetic analysis of recombinant HDR1 and HDR2 enzymes of *Populus* × *canescens* and *Picea abies*. Lineweaver-Burk plots depict data obtained from *in vitro* assays of heterologously expressed recombinant HDR1 and HDR2 enzymes of poplar **A)** and spruce **B)**. While the lines of the HDR2 measurements show similar slopes and intercepts indicating similar kinetics in both species, HDR1 showed major differences between poplar and spruce as seen in the *K*_m_- and *k*_cat_ values for the respective enzymes **C)**. The ratio of DMADP to IDP is similar for three of the four enzymes, but *Pa*HDR1 shows a remarkable increase in IDP production. Each data point represents the mean of two technical replicates from each of two separate experiments.

*HDR* sequences truncated to remove the transit peptide were cloned into *E. coli* and the heterologously expressed recombinant proteins were purified under anaerobic conditions to prevent loss of enzymatic activity. All tested proteins catalyzed the HDR reaction and showed their highest activity in a pH range of 6.0 to 6.5 at 35 to 40 °C (Supplemental Fig. S4), so all kinetic studies were conducted at pH 6.5 and 30 °C. Total product concentrations (combined amounts of DMADP and IDP) were determined with LC-MS/MS measurements to calculate initial velocities, *K*_m_ and *k*_cat_ (Supplemental Fig. S5). Calculations based on a Lineweaver-Burk plot (Fig. 3A) revealed remarkable differences between the two *Populus* × *canescens* HDRs. *Pc*HDR1 has a higher affinity to its substrate, (*E*)-4-hydroxy-3-methylbut-2-enyl diphosphate (HMBDP) than *PcHDR*2, with a *K*_m_ difference of 6.0 compared to 21.4 µM. At the same time, *Pc*HDR1 had a higher *k*_cat_ with a difference of 62.0 compared to 31.6 min^−1^, resulting in a 7-fold higher catalytic efficiency of *Pc*HDR1 versus *Pc*HDR2. The *Picea abies PaHDR*1 had a somewhat lower affinity for its substrate than *Pa*HDR2 (*K*_m_ value: 15.9 compared to 21.2 µM) and a substantially lower catalytic constant (7.8 compared to 28.0 min^−1^) and therefore a reduced catalytic efficiency (Fig. 3B).

Besides the kinetic parameters, the ratios of the DMADP and IDP products were measured by LC-MS/MS-analyses. The two *Populus × canescens* enzymes and *Picea abies* HDR2 showed similar product ratios of 1:6 to 1:9 (DMADP:IDP), respectively, whereas *Pa*HDR1 favored IDP much more with a ratio of 1:21 (Fig. 3C). There was no change in the DMADP: IDP ratio upon alterations in temperature, pH, reaction time or substrate concentration.

### Gene expression analysis and metabolic characterization of transgenic poplar with overexpressed or silenced *PcHDR*2

**Figure 4.**
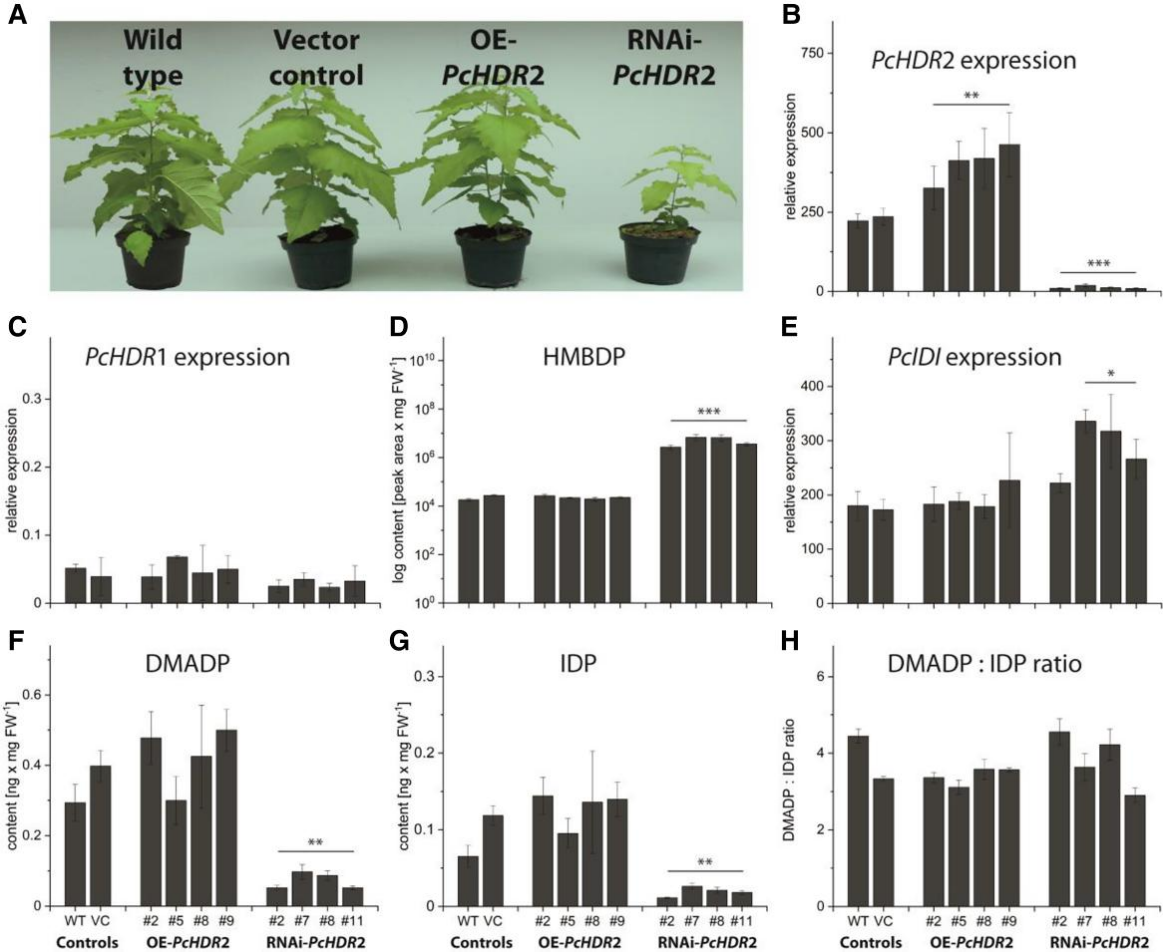
Characterization of *Populus* × *canescens* with overexpressed and silenced *PcHDR*2. Transgenic poplar plants were analyzed under greenhouse conditions when wild-type plants had reached a height of 1 m. Picture taken two weeks after transfer to the greenhouse shows plants with increased and silenced transcript levels, as well as controls. Representative plants were selected for each type of transformant that best represented its phenotype (*PcHDR*2 overexpression: line #9; RNAi-*PcHDR*2: line #2) **A)**. Analysis of *PcHDR*2 expression levels in transformants followed expectations with significant increases in overexpression lines and significant decreases in RNAi lines **B)**. Expression of *PcHDR*1 was not influenced in any of the transformants **C)**. For metabolite quantification, data were always normalized to account for the reduced biomass of the silenced lines. The HDR substrate HMBDP increased 100 to 300-fold in silenced lines compared to the controls **D)**. Expression of *PcIDI* was significantly increased in most of the silenced lines **E)**. *PcHDR*2 silencing led to a significant reduction in amounts of DMADP **F)** and IDP **G)**, but These were still present in the same ratio **H)**. Values are given as mean ± standard deviation of at least four biological replicates per line, measured in technical triplicates. Statistical analysis was performed by using Student's t-test, *** = *P* < 0.001; ** = *P* < 0.01; * = *P* < 0.05; VC, vector control; OE, overexpression.

To study the role of HDR in regulating isoprenoid biosynthesis in poplar, transgenic *Populus* × *canescens* plants were generated with overexpressed or silenced *HDR*2. We focused on *PcHDR*2 because of its more than 1000-fold higher expression relative to *PcHDR*1 (Fig. 2A and Supplemental Fig. S3, A and B). Transgenic lines in which *PcHDR*2 gene expression was reduced to about 5% of wild-type and empty vector controls displayed strong reductions in growth (50% in height, 75% in leaf area), bleached leaves, and delayed development (Fig. 4A). However, when *PcHDR*2 expression was reduced to only 10% of the controls, there were no alterations in morphology or development through metabolic changes occurred (Supplemental Fig. S6). Similarly, overexpression of *PcHDR*2 with transcript levels up to two times higher than the controls (Fig. 4B) did not lead to phenotypic differences in morphology (Fig. 4A). Notably, *PcHDR*1 gene expression was not significantly affected by either overexpression or silencing of the *PcHDR*2 gene (Fig. 4C).

To determine which enzymes or intermediates of terpenoid metabolism were affected by the manipulation of *PcHDR*2, we began with isopentenyl diphosphate isomerase (IDI), which interconverts DMADP and IDP. The expression of *PcIDI* was significantly increased in three out of four of the highly silenced *PcHDR*2 lines with altered morphology (Fig. 4E), but remained unaffected when silencing efficiency was lower (Supplemental Fig. S6B). The substrate of HDR, HMBDP, increased by a factor of about 200 in highly silenced *PcHDR*2 lines (Fig. 4D), increased by a factor of 20 in less silenced *PcHDR*2 lines (Supplemental Fig. S6E), and was unchanged in *PcHDR*2 overexpression lines (Fig. 4D). On the other hand, the end products of HDR catalysis, DMADP and IDP, were significantly reduced to about a quarter of the control values in highly silenced lines (Fig. 4, F and G), but were not altered in less silenced lines (Supplemental Fig. S6, C and D) or in overexpression lines (Fig. 4, F and G). The ratio of DMADP:IDP was not affected in any transgenic line (Fig. 4H).

**Figure 5.**
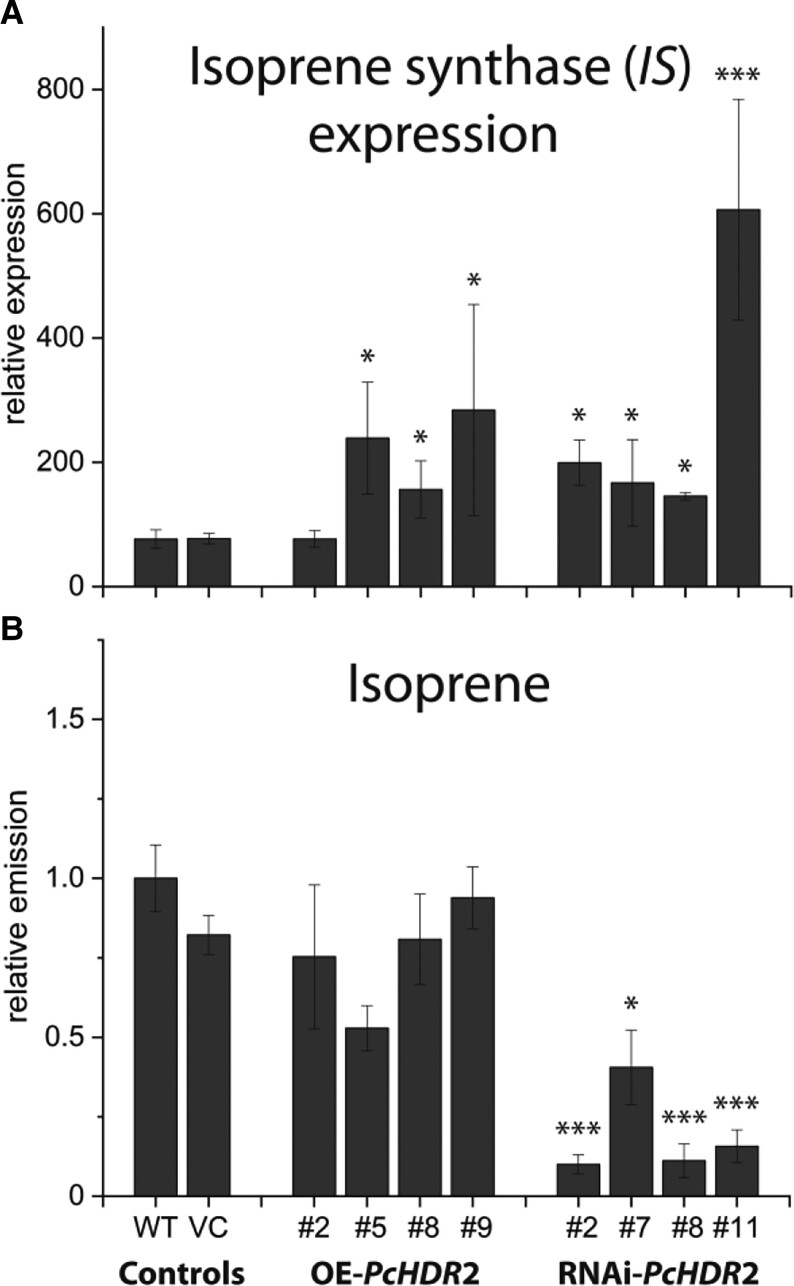
Effect of *Populus* × *canescens HDR*2 silencing on isoprene synthase expression and isoprene emission. Isoprene synthase (IS) expression significantly increased in all transgenic poplar lines, independent of their *PcHDR*2 expression **A)**. Isoprene emission, measured by performing a one-day volatile collection with normalization on plant fresh weight, was reduced in RNAi lines **B)**. Values are given as mean ± standard deviation of at least four biological replicates per line, measured in individual experiments. Statistical analysis was performed by using Student's t-test, *** = *P* < 0.001; * = *P* < 0.05; VC, vector control; OE, overexpression.

Regulation of the pool size of DMADP in poplar could be further influenced by the possibility to convert DMADP to isoprene in the one-step reaction catalyzed by isoprene synthase (IS). Therefore, *PcIS* gene expression, as well as, isoprene emission were analyzed (Fig. 5). *PcIS* expression was significantly upregulated in all transgenic overexpression and RNAi lines except for one overexpression line (Fig. 5A). In silenced lines, isoprene emission was significantly reduced, but showed no association to *PcIS* expression indicating that transcript level did not control enzyme activity under these conditions. In overexpression lines, isoprene emission was also poorly associated with *PcIS* expression (Fig. 5B).

**Figure 6.**
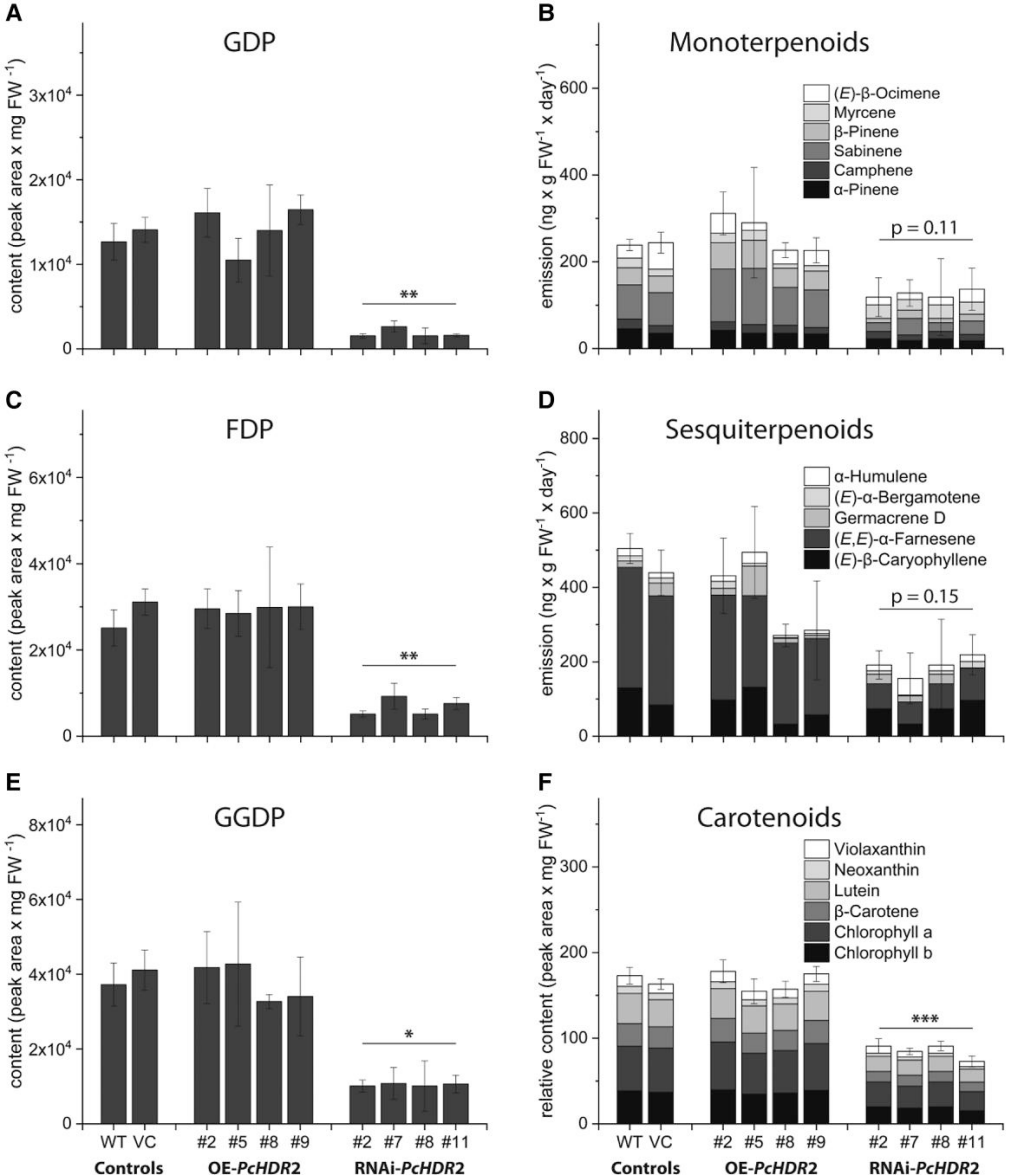
Content of prenyl diphosphate intermediates and isoprenoid products in transgenic *Populus* × *canescens* lines and controls. Prenyl diphosphates and carotenoids were extracted from fresh ground plant material, while mono- and sesquiterpenoid volatiles were collected over a 24-hour period. All prenyl diphosphate intermediates, including geranyl diphosphate (GDP; **A**), farnesyl diphosphate (FDP; **C**), and geranyl geranyl diphosphate (GGDP; **E**) were reduced in *PcHDR*2-silenced lines. Monoterpenoids **B)** and sesquiterpenoids **D)** show a tendency towards lower amounts, but this was not statistically significant. Carotenoids and chlorophylls declined significantly **F)**. Statistical analyses comparing transgenic lines with controls were performed using Student's t-test. Values are given as mean ± standard deviation of at least four biological replicates per line. *** = *P* < 0.001; ** = *P* < 0.01; * = *P* < 0.05; VC, vector control; OE, overexpression.

Based on variations in the content of DMADP and IDP in the silenced *PcHDR*2 lines, we also measured downstream terpenoid metabolites. Knock-down of *PcHDR*2 gene expression significantly decreased the levels of nearly all prenyl diphosphate intermediates, and most terpenoid end products decreased as well. In *PcHDR*2-silenced lines, GDP was reduced by more than 80% compared to the controls (Fig. 6A). However, the emission of monoterpenes (all derived from GDP) did not decline significantly (*P* = 0.11; Fig. 6B), but individual monoterpenes like sabinene and β-pinene were substantially reduced, while others like α-pinene, camphene, and myrcene were not affected. The same trend in *PcHDR*2-silenced lines was detected for C_15_ intermediates and products. FDP content was reduced significantly (Fig. 6C), while total sesquiterpene emission did not decline significantly (*P* = 0.15; Fig. 6D). Individual sesquiterpenes, such as (*E*, *E*)-α-farnesene drastically declined, while other sesquiterpenes were not affected. Among C_20_ and larger compounds, GGDP content was reduced by 70% compared to controls (Fig. 6E), while the total content of both carotenoids and chlorophylls and the levels of individual compounds were significantly reduced, as evidenced by the bleached leaves of silenced lines (Fig. 6F). In contrast to silencing, overexpression of the *PcHDR*2 gene in *Populus* × *canescens* did not cause significant changes in any of these isoprenoid intermediates or products.

### Quantification of MEP pathway intermediates, their metabolites and flux in poplar with silenced *PcHDR*2

**Figure 7.**
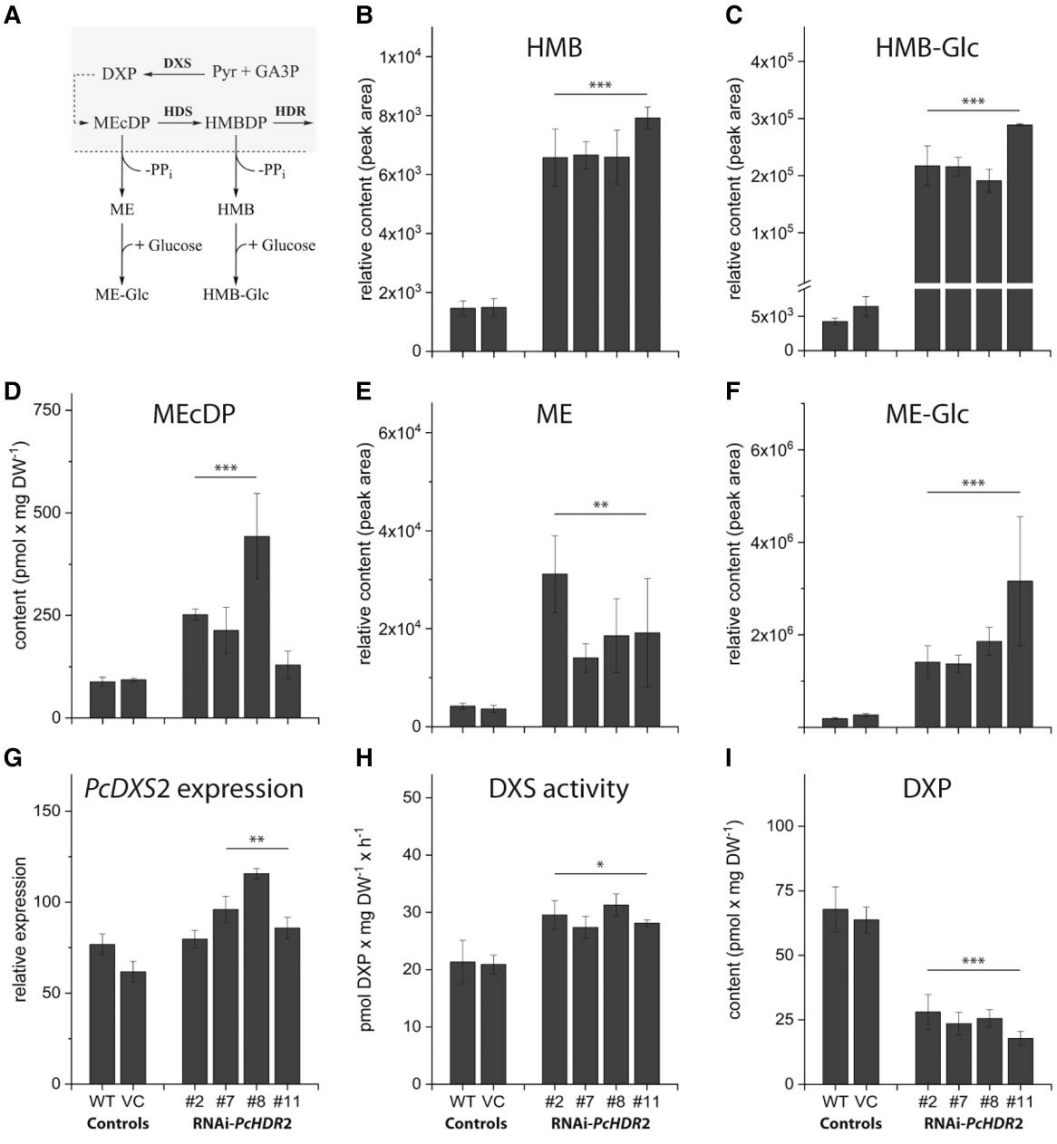
Effects of *PcHDR*2 gene silencing on methylerythritol phosphate (MEP) pathway intermediates and genes in transgenic *Populus* × *canescens* lines. Proposed conversions are depicted for the chloroplast-localized MEP pathway with a dashed line indicating the chloroplast envelope **A)**. *PcHDR*2 silencing increases the accumulation of pathway intermediates 2-C-methyl-D-erythritol-2,4-cyclodiphosphate (MEcDP) **D)** and (*E*)-4-hydroxy-3-methylbut-2-en-1-yl diphosphate (HMBDP) (Fig. 4B). HMBDP is in turn dephosphorylated to (*E*)-4-hydroxy-3-methylbut-2-enol (HMB; **B**), which is glycosylated to HMB-Glc **C)**. MEcDP gives rise to similar metabolites, the dephosphorylated 2-C-methyl-D-erythritol (ME; **E**), and the corresponding glycoside (ME-Glc; **F**). *PcHDR*2 silencing upregulates the first step of the MEP pathway, 1-deoxy-D-xylulose-5-phosphate synthase (DXS), at both the transcript **G)** and enzyme activity **H)** levels. While the concentration of 1-deoxy-D-xylulose 5-phosphate (DXP) concentration is decreased **I)**. Statistical analysis was performed by using Student's t-test of treated samples against the controls. Values are given as mean ± standard deviation of at least three biological replicates per line. *** = *P* < 0.001; ** = *P* < 0.01; * = *P* < 0.05; VC, vector control.

Altering expression of the gene encoding the last step of the MEP pathway should have metabolic consequences for earlier steps of the pathway. Here we focused our attention on *HDR*-silenced poplar lines only, since there were just minor changes in *HDR* overexpressing lines. *PcHDR*2 silencing caused more than just an accumulation of its substrate HMBDP (Fig. 4D, Supplemental Fig. S6E). There was also an increase in the amount of the next upstream intermediate, 2-*C*-methyl-D-erythritol-2,4-cyclodiphosphate (MEcDP), in three out of four silenced lines (Fig. 7D). In addition, we found that excess HMBDP was hydrolyzed to the corresponding alcohol, (*E*)-4-hydroxy-3-methylbut-2-enol (HMB) (5-fold accumulation versus controls, Fig. 7B), and its corresponding glycoside (HMB-Glc) (43-fold accumulation versus controls, Fig. 7C). Similarly, excess MEcDP was converted to give elevated levels of 2-*C*-methyl-D-erythritol (ME, Fig. 7E) and its glycoside (ME-Glc, Fig. 7F), as previously reported (Ward et al. 2012; González-Cabanelas et al. 2015).

**Figure 8.**
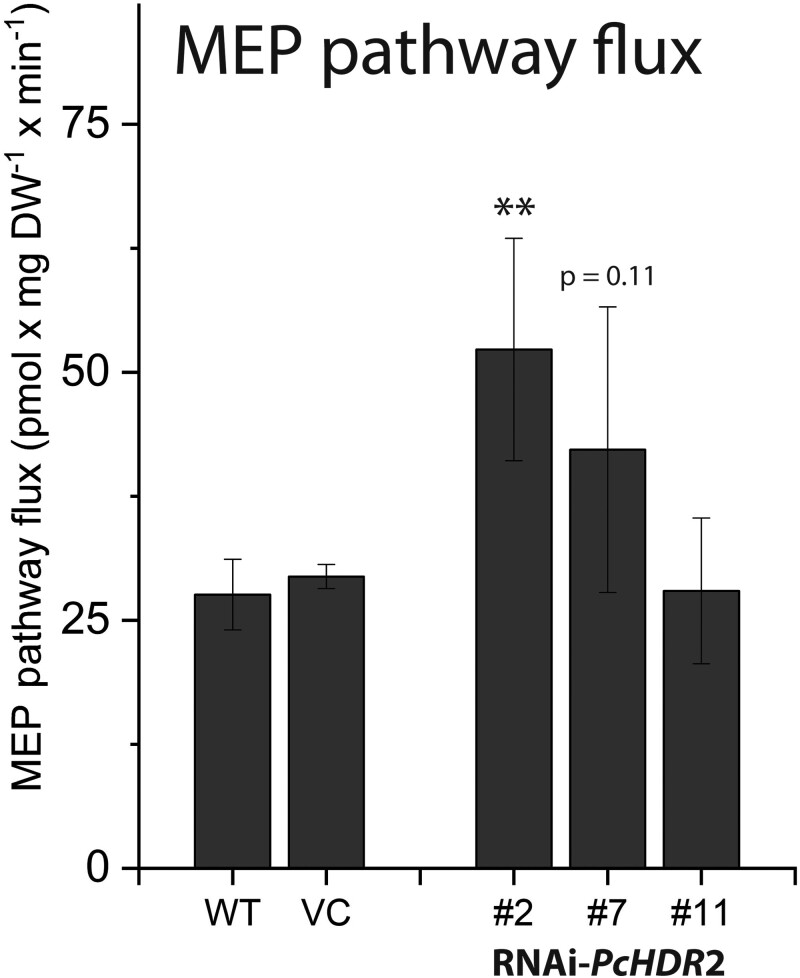
Effects of *HDR*2 gene silencing on methylerythritol phosphate (MEP) pathway flux in transgenic *Populus* × *canescens* lines. Flux was determined from fitting of time-courses of label incorporation from ^13^CO_2_ into isoprene. *HDR*2 silencing increased the flux through the pathway. Statistical analysis was performed by using Student's t-test of treated samples against the controls. Values are given as mean ± standard error of at least four biological replicates per line. ** = *P* < 0.01; VC, vector control.

The main regulator of the MEP pathway in plants is reported to be the first step, catalyzed by 1-deoxy-D-xylulose-5-phosphate synthase (DXS) (Wright et al. 2014). *PcDXS*2 expression levels were increased in three out of four *PcHDR*2-silenced lines (Fig. 7G) and DXS enzyme activity increased (Fig. 7H). In contrast, levels of the enzyme product, 1-deoxy-D-xylulose-5-phosphate (DXP) declined (Fig. 7I). These results suggested that the MEP pathway was attempting to compensate for *HDR* silencing by upregulating flux via elevated DXS activity and by diverting excess levels of the intermediates, HMBDP and MEcDP. To test this hypothesis, we measured the flux through the MEP pathway using a ^13^C-label derived from the incorporation of ^13^CO_2_ via photosynthesis through the pathway and into isoprene. Indeed, flux was increased in two of the three *PcHDR*2-silenced lines analyzed, compared to the wild-type and vector control (Fig. 8, Supplemental Table S1). The increase in MEcDP observed in the silenced *PcHDR*2 lines (Fig. 7) prompted us to analyze the level of phytohormones since this intermediate is known to serve as a stress signal in plants that modulates hormone levels (Xiao et al. 2013, Lemos et al. 2016, Mitra et al. 2021). However, the levels of jasmonate, abscisic acid, (+) and (−) jasmonic acid-isoleucine, 12-oxophytodienoic acid, and other hydroxylated forms of jasmonic acid were not different between the *PcHDR*2-silenced lines and the controls (Supplemental Table S2).

### Characterization of *PaHDR*1 and *PaHDR*2 knock-down lines in spruce

**Figure 9.**
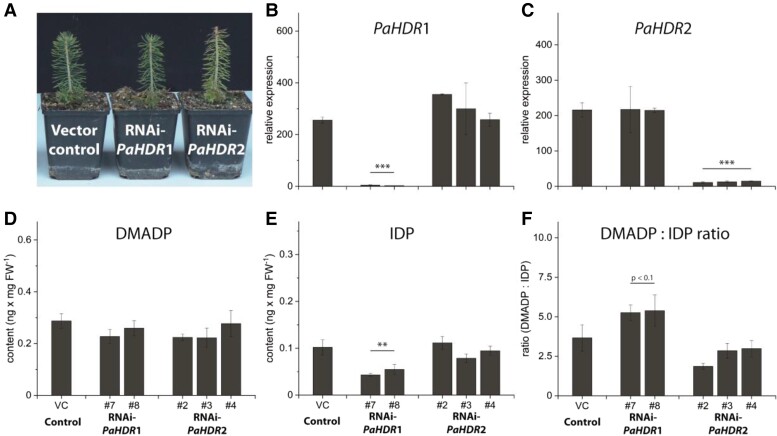
Metabolic effects of *PaHDR*1 and *PaHDR*2 gene silencing in *Picea abies*. Young saplings were transformed with an empty vector or RNAi constructs of *PaHDR*1 (2 lines) and *PaHDR*2 (3 lines). After a two-year growth period, no phenotypic differences were observed (Pictures were taken of a representative plant from line #7 for RNAi-*PaHDR*1 and line #2 for RNAi-*PaHDR*2) **A)**, but RT-qPCR analysis confirmed that HDR knock-down was specific for *PaHDR*1 **B)** and *PaHDR*2 **C)**. DMADP formation was not affected **D)**, but IDP formation was significantly affected in *PaHDR*1-silenced lines, which altered the DMADP:IDP ratio **F)**. Values are given as mean ± standard deviation of at least four biological replicates per line. Statistical analysis was performed using Student's t-test. *** = *P* < 0.001; ** = *P* < 0.01; VC, vector control.

The level of *HDR* expression in *Picea abies* was manipulated only by silencing and not overexpression since overexpression of *HDR* in poplar did not show any significant effect on isoprenoid metabolism (Fig. 6). Lines silenced in both *PaHDR*1 and *PaHDR*2 grew similarly to the vector control (Fig. 9A). Both genes were silenced by at least 95% in their respective interference lines and silencing was specific such that knock-down of one *HDR* gene had no effect on the expression of the other *HDR* (Fig. 9, B and C). In addition, *PaIDI* expression levels were not altered by *PaHDR*1 or *PaHDR*2 silencing (Supplemental Fig. S7).

*HDR* silencing in *Picea abies* did not affect most of the metabolites analyzed. DMADP levels were unchanged after *PaHDR*1 or *PaHDR*2 silencing (Fig. 9D), while IDP levels did decline upon *PaHDR*1 silencing, but were not altered by *PaHDR*2 silencing (Fig. 9E). Thus, the DMADP:IDP ratio was elevated when *PaHDR*1 was silenced, but not *PaHDR*2 (Fig. 9F). The levels of the prenyl diphosphates GDP, FDP and GGDP were also unaffected by *HDR* silencing (Supplemental Fig. S8A, B, C). In addition, the corresponding terpenoid end products were generally not altered except that monoterpenoid emission was substantially reduced in one of the three *PaHDR2*-silenced lines (Supplemental Fig. S8D). Sesquiterpenes and diterpenes, as well as carotenoids, were not significantly affected by reduced *HDR* expression levels (Supplemental Fig. S8E, F, G).

## Discussion

HDR is the last enzyme of the MEP pathway, producing DMADP and IDP, which serve as essential building blocks for isoprenoid biosynthesis. Although the flux of the MEP pathway is reported to be controlled mainly by DXS activity, which is modulated at different regulatory levels (Banerjee et al. 2013; Hemmerlin 2013), HDR may influence terpenoid end product distribution by controlling the ratio of DMADP to IDP available. The formation of larger terpenoids requires a greater proportion of IDP units compared to DMADP. Therefore, in this study, we investigated HDR in two species of plants with differing terpenoid product profiles. The gymnosperm Norway spruce (*Picea abies*) produces large amounts of monoterpenes (C_10_), sesquiterpenes (C_15_), and diterpenes (C_20_) (Martin et al. 2002; Keeling and Bohlmann 2006; Schmidt et al. 2010; Schmidt et al. 2011; Nagel et al. 2022), but only low amounts of isoprene (C_5_) (Perreca et al. 2020). On the other hand, the gray poplar (*Populus* × *canescens*) is an angiosperm that emits large amounts of isoprene (C_5_) and lower levels of monoterpenes (C_10_) and sesquiterpenes (C_15_) (Ghirardo et al. 2014; McCormick et al. 2014). Both species, like other green plants, produce carotenoids and the phytol side chain of chlorophyll, which are both derived from a C_20_ isoprenoid intermediate.

We found that *Picea abies* has two distinct HDR isoforms, one producing relatively more IDP for primary isoprenoids (carotenoids and chlorophylls formed from C_20_ precursors) and one producing relatively more DMADP for the specialized isoprenoid resin compounds in this species, which are formed from C_10_, C_15_, and C_20_ precursors. In contrast, *Populus* × *canescens* has only one dominant isoform, which produces relatively high levels of DMADP, but this species makes mostly primary isoprenoids (formed from C_20_ precursors). These require a greater supply of IDP rather than DMADP, and the remaining DMADP appears to be emitted as isoprene.

### *(E)*-4-Hydroxy-3-methylbut-2-en-1-yl diphosphate reductase (*HDR*) genes are differentially expressed in poplar and spruce

Plants possess a variable number of *HDR* genes, which appears to correspond with the complexity of their terpenoid profiles. *Arabidopsis thaliana* for example, harbors only a single *HDR* copy (Cheng et al. 2017) and produces only limited amounts of terpene natural products, chiefly as volatiles or trace root consistutents (Chen et al. 2003; Vaughan et al. 2013; Wang et al. 2016). Plants containing two *HDR* genes, including *Artemisia annua*, *Ginkgo biloba*, *Picea sitchensis*, *Pinus taeda*, and *Cucumis melo* (Carrier et al. 1998; Portnoy et al. 2008; Ma et al. 2017; Bongers et al. 2020) typically make large amounts of various terpenoid natural products, such as sesquiterpenes (*A. annua*), diterpenes (*G. biloba*) and monoterpenes and diterpenes (*Picea sitchensis*, *Pinus taeda*). We found that both *Populus* × *canescens* and *Picea abies* contain two catalytically active HDR isoforms, one of which could be associated with formation of terpenoid defenses or other natural products.

In *Populus* × *canescens*, *PcHDR*1 is expressed in much lower amounts than *PcHDR*2, but is represented more in roots than in other organs (Fig. 2A), suggesting a different role than *PcHDR*2. Gene expression data from *Populus trichocarpa* also suggested different roles for the two *HDR* genes. Expression of *PtHDR*2, but not *PtHDR*1, is induced by herbivore attack or oomycete infestation, suggesting a function of *PtHDR*2 in the production of defense metabolites or signals under biotic stresses (Supplemental Fig. S3, A and C). On the other hand, treatment with jasmonic acid caused both *PtHDR*1 and *PtHDR*2 to be expressed at higher levels than their untreated controls (Supplemental Fig. S3, B and D). More experiments are needed to distinguish the roles of the two popular *HDR* genes.

In *Picea abies*, *PaHDR*2 is the dominant form in stems and roots, which are largely non-photosynthetic. Moreover, *PaHDR*2 expression is much more inducible in needles and stems than *PaHDR*1 after treatment with the defense hormone methyl jasmonate (Supplemental Fig. S3E). These results suggest a role for *PaHDR*2 in the formation of the abundant terpenoid-based resins of stems and roots. This gene may also be involved in the formation of terpenoid resins in needles where it is expressed in a similar level as *PaHDR*1. In this scenario, *PaHDR*1 would then encode an enzyme involved in formation of C_5_ units for the primary isoprenoids, carotenoids, and chlorophylls. Similar roles have been suggested for the two HDR proteins of *Picea sitchensis* based on in vivo measurements after expression in *Escherichia coli* (see next section) (Bongers et al. 2020).

The origin of the two *HDR* genes in these species is unclear, but based on our phylogeny (Supplemental Fig. S2A), duplication likely occurred independently in the phylogenies of *Populus* and *Picea*. In gymnosperms, this duplication must have happened before the *Ginkgo* line split off from that of conifers such as *Picea* (Xi et al. 2013). In angiosperms, loss of *HDR* genes seems to be ongoing in several species. For example, in rice (*Oryza sativa*) *OsHDR*2 has a mutation in the first exon leading to the formation of a stop codon, which prevents the expression of a functional enzyme (Lee et al. 2022). A similar loss of activity has also been described for one HDR isoform in melon (*Cucumis melo*). Thus, *HDR* in angiosperms seems to have undergone a duplication followed by losses to give lineages with a single *HDR* as a derived trait (Saladié et al. 2014).

### Spruce HDR enzymes May Have distinct roles in primary and specialized metabolism

Recombinant proteins of HDR1 and HDR2 from both *Populus* × *canescens* and *Picea abies* were catalytically active in in vitro assays after expression in *E. coli* and extraction under nitrogen atmosphere to avoid oxidation and decomposition of the iron-sulfur cluster. This [4Fe-4S] cluster, previously described for *Escherichia coli* IspH (Graewert et al. 2009), catalyzes the reduction and dehydroxylation of HMBDP to DMADP and IDP. The catalytic properties of the four enzymes expressed were in part quite different, leading to differences in the ratio of DMADP:IDP produced. For *Populus* × *canescens Pc*HDR1 and *Pc*HDR2 and *Picea abies Pa*HDR2, the ratios range from 1:6 to 1:9. However, *Pa*HDR1 displayed a DMADP:IDP ratio of 1:21 (Fig. 3C). Previous publications on the recombinant *Ec*HDR from *E. coli* expressed in vitro reported DMADP:IDP ratios of 1:4 to 1:6 (Adam et al. 2002; Rohdich et al. 2003). In plants, prior work on a recombinant *Gb*HDR from *G. biloba* assayed in vitro demonstrated that the enzyme yields a mixture of DMADP and IDP in a ratio of 1:15 (Shin et al. 2015) based on the amount of dephosphorylated and volatile alcohols prenol and isoprenol, respectively. This ratio is in the range of the plant DMADP:IDP ratios reported in this study. However, due to the uncertainties associated with quantitative dephosphorylation and the volatility of the products, this method is likely less accurate than the direct quantification of DMADP and IDP by LC-MS as performed in the present study. These characterizations of plant HDR enzymes employing a protocol for purification and analysis of this oxygen-sensitive enzyme under anaerobic conditions as well as an analytical method to resolve the two products and quantify them directly.

The large potential diversity in product profiles of plant HDR enzymes was noted by (Bongers et al. 2020). These authors assayed the in vivo activity of various plant HDRs expressed in *E. coli* by determining the intracellular DMADP and IDP concentrations achieved. These measurements were performed in the presence of the native *E. coli* HDR, as well as an additional poplar DXS and an added bacterial lycopene pathway to prevent toxic build-up of prenyl diphosphates. Despite these big differences of the approach to our in vitro assays of recombinant HDR, the results of Bongers et al. show a very similar trend to ours. Measuring the two HDRs of *Picea sitchensis*, they found *Ps*HDR1 to produce a DMADP:IDP ratio of 0.5:1 in vivo, one of the lowest such ratios of any of the HDR enzymes they studied. We studied *Pa*HDR1 of *Picea abies*, whose sequence is identical to that of *Ps*HDR1, and found an in vitro ratio of 1:21. While this ratio seems superficially quite different from the in vitro value obtained by Bongers et al. for *Ps*HDR1, it was also the lowest ratio obtained for any of the spruce and popular enzymes we studied. A similar parallel is evident for spruce HDR2. *Ps*HDR2 gave a DMADP:IDP ratio of 10:1 in vivo (Bongers et al. 2020), one of the highest such ratios measured by these authors. Meanwhile, in our hands the *Pa*HDR2 gave a DMADP:IDP ratio of 1:6 in vitro, the highest ratio we observed among the spruce and poplar enzymes in this study.

Hence, though the specific in vitro DMADP:IDP ratios we measured vary greatly from those determined in vivo by Bongers et al., the relative order of *Picea* enzymes in both our characterizations are similar: HDR1 produces more IDP and HDR2 produces more DMADP. This trend is supported by the results of silencing *PaHDR*1, which caused a significant decrease in the internal pools of IDP, but not DMADP (Fig. 9, D and E). The distinction between the two *Picea* enzymes defines specific roles for each that are supported by gene expression data. By producing a lower ratio of DMADP:IDP, HDR1 is well suited for the MEP pathway in cellular compartments where an abundant supply of GGPP is needed for larger terpenoids, such as the chlorophylls and carotenoids made in chloroplasts. We found the *PaHDR*1 gene to be strongly expressed in a constitutive manner in spruce needles, but in only low amounts in stems and roots. On the other hand, *Pa*HDR2 produces relatively more DMADP and so is well suited to support the MEP pathway in cellular compartments where an excess of GDP and FDP is needed for forming smaller terpenoids, such as the monoterpenes and sesquiterpenes of spruce oleoresin made in resin duct epithelial cells in stems and roots. Consistently, we found the *PaHDR*2 gene to be more dominant than *PaHDR*1 in stems and roots, and to be induced by treatment with the defense hormone methyl jasmonate. Together, these results point to distinct roles for HDR1 (primary metabolism) and HDR2 (specialized metabolism). However, specific knock-down of *PaHDR*1 in needles did not reduce primary metabolites such as carotenoids or chlorophylls. Possible explanations are compensation by *Pa*HDR2 at the protein level, or transfer of isoprenoid intermediates from other tissues. These mechanisms should be examined in further research. Future investigation should also determine what features of the HDR1 and HDR2 proteins themselves are associated with different DMADP:IDP ratios. Current sequence comparisons (e.g. Supplemental Fig. S1) do not provide much information on this point.

The ratio of DMADP:IDP available in plants also depends on the enzyme isopentenyl diphosphate isomerase (IDI), which equilibrates between these C_5_ diphosphates favoring DMADP by a ratio of 2:1 to 7:1 based on in vitro measurements (Luetzow and Beyer 1988; Ramos-Valdivia et al. 1997). These in vitro characteristics of IDI result in an in vivo DMADP:IDP ratio of approximately 2:1 to 4:1, according to a survey of plant species (Krause et al. 2020). Thus, if there is an excess of DMADP over IDP in vivo, an enzyme such as HDR1 that supplies more IDP, based on in vivo (Bongers et al. 2020) and in vitro (our) measurements, may be important in supporting the formation of GGDP needed for chlorophyll and carotenoid biosynthesis in photosynthetic tissue.

### HDR regulates terpenoid formation in different ways in spruce compared to poplar

The major motivation for our study was to compare the role of HDR in terpenoid biosynthesis in two woody plant species with different terpenoid profiles. We hypothesized that the properties of HDR might be tailored to support different outputs of the pathway products, DMADP and IDP, depending on the various terpenoid end products formed. In both *Picea abies* and *Populus* × *canescens*, the major MEP pathway products, carotenoids and the chlorophyll side chains, are produced from the intermediate GGDP (C_20_, requires 1 mole DMADP and 3 moles IDP). *Picea abies,* however, but not *Populus* × *canescens*, produces high concentrations of an oleoresin composed of monoterpenes from GDP (C_10_, requires 1 mole DMADP and 1 mole IDP), sesquiterpenes from FDP (C_15_, requires 1 mole DMADP and 2 moles IDP) and diterpenes from GGDP. In *Populus* × *canescens*, however, there are no major terpenoid natural products accumulating in the leaves. Instead, isoprene is formed in high amounts from DMADP (C_5_), requiring no IDP.

The two species seem to employ different ways to regulate the production of these terpenoids. In *Picea abies*, as discussed in the last section, there are two HDR enzymes with different biochemical properties, different locations, and likely different roles in primary versus specialized metabolism. The gene encoding *Pa*HDR1 is constitutively expressed and only present in needles. This enzyme has the lowest DMADP:IDP ratio of any of the HDR enzymes we characterized in this study. With its preference for producing IDP, *Pa*HDR1 seems tailored to the maximize formation of the largest intermediate, GGDP (C_20_, requires 1 mole of DMADP, 3 moles of IDP), used to make carotenoids and chlorophylls, the essential terpenoid products of green tissue. *Pa*HDR2, on the other hand, is constitutively expressed in stems and roots, as well as needles, and is also induced by the defense hormone methyl jasmonate in needles and stems, a pattern that fits the occurrence of terpenoid resin ducts in spruce. *Pa*HDR2 forms a much higher ratio of DMADP:IDP than *Pa*HDR1 and so fits the requirements of making spruce resin better, which utilizes on average a greater supply of DMADP than carotenoid or chlorophyll side chain biosynthesis. Spruce resin contains monoterpenes, sesquiterpenes, and diterpenes, arising respectively from GDP (C_10_, requires 1 mole DMADP and 1 mole IDP), FDP (C_15_, requires 1 mole DMADP and 2 moles IDP) and GGDP (C_20_, requires 1 mole DMADP and 3 moles IDP).

In *Populus* × *canescens*, the expression of HDR-encoding genes was dominated by *PcHDR*2 in all organs measured. The encoded enzyme appears to be involved in making all of the terpenoid end products detected in this species (isoprene, monoterpenes, sesquiterpenes, carotenoids and chlorophylls) since all decline significantly on *PcHDR*2 silencing (Figs. 5 and 6). Yet the *Pc*HDR2 enzyme makes a high ratio of DMADP:IDP even though its major products, the carotenoids and chlorophyll side chains, are formed from GGDP (C_20_, requires 1 mole DMADP and 3 moles IDP). The formation of principally carotenoids and chlorophylls from MEP pathway products should leave a large excess of DMADP, which may account for the high rate of isoprene release in *Populus* × *canescens*. Isoprene formation results from the action of a single enzyme that converts DMADP directly to isoprene via dephosphorylation, double-bond rearrangement, and deprotonation. Could the formation of isoprene function as a way to reduce the imbalance between the DMADP and IDP formed from the MEP pathway and that utilized by terpenoid biosynthesis?

### HDRs with a high DMADP:IDP ratio may help explain the formation of isoprene

The role of isoprene in plants has been studied extensively for many years since it is released in detectable amounts by 20% of the plant species in the world and is the most abundant hydrocarbon released into the atmosphere from the earth's vegetation (Sharkey and Yeh 2001; Loreto and Fineschi 2015). This research provides some support for the function of isoprene in resistance to abiotic stresses due to its chemical and physical properties. Isoprene can quench reactive oxygen species (Loreto et al. 2001; Vickers et al. 2009) and increase thermotolerance either by strengthening membranes (Singsaas et al. 1997) or reducing membrane temperature by evaporation (Pollastri et al. 2014). Nevertheless, recognition of the small amounts of isoprene actually present in plants (Harvey et al. 2015) has now led to a broad consensus that this molecule likely acts as a stress signal in plants by altering gene expression (Zuo et al. 2019), protein abundance (Vanzo et al. 2016) and metabolite content (Ghirardo et al. 2014). Yet, based on consideration of the DMADP:IDP ratios produced by HDR, isoprene could just as well be considered a vehicle for removing excess DMADP.

The idea that isoprene emission is a metabolic “safety valve” for breaking down high amounts of DMADP while recovering the pyrophosphate moiety was proposed almost 20 yr ago (Rosenstiel et al. 2004) and even suggested to regulate the balance between primary and specialized terpenoid metabolism (Owen and Peñuelas 2005). While it is not surprising that a volatile metabolite such as isoprene has come to be employed by plants as a signal, its original purpose may have been just to remove excess DMADP. Plants with an HDR that makes a high ratio of DMADP:IDP could alleviate a build-up of DMADP by forming isoprene or perhaps volatile monoterpenes. Bongers et al. (2020) discovered a strong association between plant species that contain an HDR furnishing a high ratio of DMADP:IDP and those that release isoprene and other volatile terpenes. These authors make a strong argument that such HDR enzymes might have been specifically selected for volatile formation. On the other hand, if these enzymes form high DMADP:IDP ratios for other reasons, such as to optimize enzyme performance, isoprene formation can be viewed as a way to compensate for the excess DMADP produced. Future research should study additional examples of HDR enzymes in isoprene-emitting and non-emitting plant species while examining the DMADP:IDP ratios formed in vitro and present in vivo. In this context, it would also be interesting to study the influence of IDP isomerase (IDI). As mentioned above, high activities of IDI could alleviate the build-up of DMADP and supply additional IDP to form the GGDP needed to produce carotenoid and chlorophyll pigments.

### Perturbation of *HDR* expression and MEP pathway metabolite levels is counteracted by homeostatic mechanisms to maintain pathway flux

The MEP pathway is essential for producing the DMADP and IDP in plastids used for the formation of isoprenoids such as carotenoids, chlorophyll side chains, various plant hormones, and a host of natural products (Rodríguez-Concepción 2006). Thus, it is not surprising that when the expression of pathway genes, such as *HDR*, or levels of intermediates are altered there are mechanisms to maintain the normal operation of the pathway. Separate silencing of each of the two *Picea abies HDR* genes resulted in transcript levels less than 5% of wild-type levels in each case (Fig. 9). However, no morphological changes were observed, and only minor effects on metabolites were detected. Hence, it appears that the reduced expression of one *HDR* copy can be mostly compensated for by the second gene in this species, even though there was no measurable increase in expression. Similarly, in *Artemisia annua*, which harbors two *HDR* isoforms, there were no drastic morphological changes when one gene was silenced (Ma et al. 2017). Both *Arabidopsis thaliana* and *Nicotiana benthamiana*, on the other hand, contain only one copy of *HDR*. In these species, silencing resulted in reduced growth (Page et al. 2004; Hsieh and Goodman 2005). *Populus* × *canescens* also has two *HDR* genes, but in leaves only *PcHDR*2 is expressed at a substantial level. Silencing *PcHDR*2% to 5% of wild-type levels resulted in substantial growth reductions as well as declines in the pools of DMADP, IDP and other isoprenoid intermediates and products (Figs. 4 and 6).

Silencing of *PcHDR*2 also caused the build-up of the upstream MEP pathway intermediates, MEcDP and HMBDP, in *Populus* × *canescens*. These intermediates were also found to be dephosphorylated and glucosylated to generate ME-Glc and HMB-Glc, respectively, reactions previously reported in *A. thaliana* (Ward et al. 2012; González-Cabanelas et al. 2015) (Fig. 7). Such reactions may represent detoxification mechanisms for over-accumulating metabolites containing a diphosphate group, since it was reported that increased concentrations of IDP reduce the survival and growth of genetically modified bacteria due to toxicity of the diphosphate moiety (George et al. 2018). Recycling of inorganic diphosphate might also be crucial for maintaining the rate of energy-generating metabolic reactions (Weiner et al. 1987). Under some conditions, however, accumulation of MEcDP might be beneficial to plants by preventing chloroplast damage under high light conditions due to the ability of this intermediate to scavenge hydroxyl radicals (Rivasseau et al. 2009). Hence, the rate of HDR activity could be modulated so that the pathway can serve this additional function.

MEcDP is also known to act as a plastid-to-nucleus signal involved in biotic and abiotic stress responses (Xiao et al. 2013) that might also contribute to the regulation of MEP pathway enzymes (Mitra et al. 2021). MEcDP was especially described to modulate salicylic acid (SA) and jasmonic acid (JA) biosynthetic genes (Lemos et al. 2016). Therefore, jasmonate levels were quantified in *PcHDR*2-silenced *Populus* × *canescens* to examine if MEcDP accumulation in these lines influences hormone levels. However, jasmonates were not altered in *PcHDR*2-silenced poplar (Supplemental Table S2), consistent with the finding that SA induction by MEcDP in *A. thaliana* does not lead to antagonism with JA signaling (Onkokesung et al. 2019). Since poplar and *A. thaliana* show other differences in JA-SA interaction (Ullah et al. 2022), more research is required to examine the relationship between the MEP pathway and hormone signaling in poplar and other species.

To maintain MEP pathway flux, the silencing of *PcHDR*2 in *Populus* × *canescens* was also compensated for by the upregulation of 1-deoxy-D-xylulose-5-phosphate synthase (DXS), the first step of the MEP pathway. DXS is known to be the rate-limiting step of the MEP pathway in *A. thaliana* (Wright et al. 2014), and may be regulated at the levels of transcription (Phillips et al. 2007; Saladié et al. 2014), substrate supply (Banerjee and Sharkey 2014) or feedback inhibition by the pathway end-products (the HDR products), DMADP and IDP (Banerjee et al. 2013). In our *HDR*-silenced *Populus* × *canescens* lines, both *PcDXS*2 expression and *Pc*DXS enzyme activity were significantly increased (Fig. 7, G and H), consistent with regulation at the transcriptional level and by feedback inhibition (lower IDP and DMADP levels (Fig. 4, F and G) should result in reduced feedback inhibition). This suggests the operation of a mechanism to increase MEP pathway flux and indeed, increased flux was observed for two out of the three HDR-silenced lines (Fig. 8). Given the importance of the MEP pathway in plant metabolism, future research is likely to detect other ways to ensure its homeostasis under a wide range of growing conditions.

The formation of plant isoprenoids as a whole has a unique homeostatic mechanism in that all plants possess two pathways for producing the C_5_ prenyl diphosphate intermediates: the MEP pathway in plastids and the mevalonate (MVA) pathway localized in the cytosol with inter-pathway exchange of intermediates shown in a number of cases (Dudareva et al. 2005; Skorupinska-Tudek et al. 2008; Henry et al. 2018). Hence, if one isoprenoid pathway is functioning sub-optimally, the second pathway could theoretically compensate. It is generally assumed that FDP and sesquiterpenes are MVA-derived (Rodríguez-Concepción 2006). However, we found that *HDR*2 silencing in *Populus* × *canescens* reduced not only the plastidial isoprenoids derived from the MEP pathway, such as carotenoids, chlorophylls, monoterpenes, and diterpenes, but also FDP and sesquiterpenes (Fig. 6). This suggests that the MEP pathway in poplar leaves might be a source of either DMADP or IDP or both for sesquiterpene formation. And, the lack of increase in FDP and sesquiterpenes also suggests that the MVA pathway is not activated in *PcHDR*2-silenced plant lines under the conditions we investigated. Unfortunately, we did not measure any other typical MVA pathway products in *PcHDR*2-silenced lines, such as sterols, phylloquinone, ubiquinone, polyprenols, and dolichols, so we do not know whether they are also formed with precursors from the MEP pathway. Nevertheless, this and other findings should help motivate researchers to continue investigating the source pathway for isoprenoids in a greater range of plant species, organs, and developmental stages, to determine the extent of cross-talk between the pathways.

## Materials and methods

### Plant cultures

Gray poplar (*Populus × canescens*), (clone INRA 7171-B4) and western balsam-poplar (*Populus trichocarpa*), (clone “Muehle-Larsen”; *P*&P Baumschule, Beverstedt, Germany) were propagated and grown in a greenhouse (24 °C, 60% relative humidity, 100 µmol m^−2^ s^−1^ PPFD, and 16 h/8 h light/dark) in a 1:1 mixture of sand and soil (Klasmann-Deilmann, Geeste, Germany) until they reached a height of either 1 m (*P. × canescens*) or 40 cm (*P. trichocarpa*). After whole plant measurements, such as volatile collection, plant material was flash-frozen in liquid nitrogen for further analysis. For analyzing the leaves, leaf no. 9 was chosen according to the leaf plastochron index (LPI) (Frost et al. 2007). For stem analyses, a mixture of 2 cm long sections from the top, middle and bottom stem were analyzed, and for roots, an equal mixture of tips and main root parts were analyzed. Four plants per transgenic line were used for wild-type and transgenic poplar, except for empty vector controls, which were represented by three lines with four plants each.

### Accession numbers

Sequence data from this article can be found in the GenBank/EMBL data libraries under accession numbers (*Pc*HDR1 (*Populus* × *canescens* HDR1; XP_002313816.1, *Pc*HDR2 (*Populus* × *canescens* HDR2; XP_002305413.1, *Pa*HDR1 (*Picea abies* HDR1; BT115538.1), *Pa*HDR2 (*Picea abies* HDR2; MA_105092g0010)

For jasmonic acid treatment, poplar plants were irrigated with 250 µmol ± jasmonic acid (Cayman Chemical Company, Ann Arbor, MI, USA) dissolved in water on two successive days, and on the third day, leaves (LPI No. 3 to 6) were harvested, flash-frozen with liquid nitrogen, and stored at −80 °C until further sample processing. Here, six wild-type plants were used per treatment or as controls.

Norway spruce (*Picea abies*) saplings propagated from clone 3369-Schongau (Samenklenge and Pflanzgarten, Laufen, Germany) were used for methyl jasmonate (MJ) (Merck, Darmstadt, Germany) treatment, and an embryogenic culture of clone 186/3c VIII (kindly provided by Harald Kvaalen, Norwegian Institute of Bioeconomy Research, Ås, Norway) was used for making transgenic lines. Both clones were grown under alternating summer and winter conditions. Summer conditions consisted of standard soil under a 21 °C-day/16 °C-night temperature cycle, controlled light conditions (16 h per day at 150 to 250 *μ*mol, obtained from a mixture of cool-white fluorescent and incandescent lamps), and a relative humidity of 70% in a climate chamber (Weiss Technik GmbH, Reiskirchen, Germany). For the 8-week winter period, the temperature during day and night was constant at 6 °C, with light for only 8 h per day.

For characterization, two-year-old transgenic saplings with a size of 7 ± 5 cm were detached 0.5 cm above the ground and flash-frozen in liquid nitrogen. Needles were separated by carefully breaking them from the frozen stem. Roots were separated from the remaining stem tissue and washed to remove soil particles. Needles, stems and roots were ground separately to a fine powder and stored at −80 °C. Four plants were sampled for each transgenic spruce line, while empty vector controls were represented by two lines with four plants each. For MJ treatment, four biological replicates of three-year-old plants were sprayed with 1 L of 1 mM MJ solution. Samples were collected before spraying and after 2 and 6 d of treatment.

### *HDR*1 and *HDR*2 sequences from poplar and spruce

Based on available sequences from *Populus trichocarpa* and white spruce (*Picea glauca),* HDR1 and HDR2 amino acid sequences from *Populus × canescens* and *Picea abies* were obtained from the NCBI and congenie.org databases. NCBI gene accession/congenie numbers for *Populus* × *canescens* were XP_002313816/Potri.004G150400 and XP_002305413/Potri.009G111600 for *Pc*HDR1 and *Pc*HDR2, respectively; sequences were verified by subcloning and sequencing to be identical to those of *Populus trichocarpa*. The NCBI gene accession number for *Picea glauca* HDR1 was BT115538, which was verified by sequencing to be identical to *Pa*HDR1. The congenie.org accession number for *Pa*HDR2 was MA_105092g0010.

The DNAStar Lasergene program version 13.0 (MegAlign) was used to align and to calculate the deduced amino acid sequences of each full-length cDNA or of known sequences from other gymnosperms and angiosperms. The amino acid alignment was conducted by use of ClustalW (gonnet 250 matrix, gap penalty 10.00, gap length penalty 0.20, delay divergent sequences 30%, gap length 0.10, DNA transition weight 0.5). The same software was used to visualize the phylogenetic tree (Supplemental Fig. S1). Screening for intracellular localization sequences used web-based tools like ChloroP, TargetP, and SignalP (http://www.cbs.dtu.dk), which revealed that all HDR1 and HDR2 proteins possess putative chloroplast targeting sequences.

### Cloning and heterologous expression

Total RNA was extracted from frozen and ground *Populus × canescens* and *Picea abies* plant material using the InviTrap Spin Plant RNA Mini Kit (Invitek, Berlin, Germany) and cDNA libraries were prepared using the SuperScript III First-Strand Synthesis SuperMix (Thermo Fisher Scientific, Waltham, MA, USA). For expression of HDR proteins, truncated sequences were used in a gateway cloning system (Thermo Fisher Scientific) using appropriate primers (Supplemental Table S3). Gel-purified genes of interest were introduced into pDONOR207 vectors followed by transformation into One Shot™ TOP10 competent *E. coli* (Thermo Fisher Scientific). Positive clones were verified by Sanger sequencing, further subcloned into a pDEST15 expression vector (Thermo Fisher Scientific) and transformed into BL21-AI™ One Shot™ competent *E. coli* (Thermo Fisher Scientific). These were used directly for inoculation of a 12 mL preculture of LB medium, incubated for 72 h at 18 °C and 220 rpm, and further used to inoculate 100 mL LB. The medium was supplemented with 1 mM L-cysteine and ferric ammonium citrate (30 µg × mL^−1^) to ensure proper iron supply for the formation of the iron-sulfur-cluster of the HDR according to (Graewert et al. 2009). The culture was grown until OD_600_ reached 0.6, induced with 0.2% (w/v) L-arabinose and grown overnight at 18 °C and 220 rpm. The whole procedure was performed with two technical replicates.

### Purification of the recombinant HDR protein with exclusion of oxygen

The induced culture was harvested by centrifugation at 3000 × g, 4 °C for 10 min and the cell pellet was covered with argon. All further steps were performed in a glovebox filled with nitrogen gas (GS GLOVEBOX Systemtechnik GmbH, Malsch, Germany). Oxygen level was monitored using a BW clip real-time O_2_ sensor (BW Technologies, Calgary, Canada). The pellet was resuspended in 3 mL of 25 mM MOPSO (β-hydroxy-4-morpholine-propanesulfonic acid) buffer supplemented with 25 mM MgCl_2_, 50 mM KCl and 10% (v/v) glycerol and sonicated on ice for 3 min, 2 × 10% cycle with 60% power using an sonoplus HD2070 ultrasonic homogenizer (Bandelin Electronic, Berlin, Germany). The disrupted cell suspension was centrifuged for 15 min at RT and 14,000 × g and the supernatant was further purified using 3 mL Pierce™ Glutathione Spin Columns (Thermo Fisher Scientific) according to the manual. To ensure an oxygen-free environment, the columns were additionally flushed with 1 mL 10 mM sodium dithionite between two equilibration steps of wash buffer. Binding of the enzyme to the column took place while the column was incubated horizontally on ice for 2 h and gently shaken every 30 min. For enzyme assays, the first two fractions eluted, 1 mL each, were combined. Protein concentration was determined via a Bradford Assay using the Quick Start™ Bradford Protein Assay kit (Bio-Rad Laboratories GmbH, Hercules, CA, USA). A calibration curve was prepared with Bovine Serum Albumin (BSA) standards by measuring three independent technical replicates.

### HDR enzyme assays under anaerobic conditions

The 2 × assay buffer consisted of a mixture of 100 mM MES (2-(N-morpholino)ethanesulfonic acid), 100 mM HEPES (4-(2-hydroxyethyl)-1-piperazineethanesulfonic acid), 100 mM CHES (2-(cyclohexylamino)ethanesulfonic acid), 50 mM MgCl­_2_, 100 mM NaCl and 20% (v/v) glycerol according to (Shin et al. 2015). The assays were performed in 200 µL portions with 100 µL 2 × assay buffer, 1 mM methyl viologen, 3 mM sodium dithionite, 0.5 µg HDR protein, and variable amounts of HMBDP substrate (*(E)*-4-hydroxy-3-methylbut-2-enyl diphosphate lithium salt). All components were purchased from Merck, Darmstadt, Germany. Assays were optimized regarding temperature and pH. After testing values of 8 to 50 °C and pH 3.5 to 9.5, 35 °C and pH 6.5 were considered as optimal reaction conditions and applied in enzyme assays for measuring kinetic parameters. All reactions were stopped by adding 100 µL chloroform and centrifuged for 5 min, at 4 °C and 14,000 × g. The aqueous phase was transferred into new vials and stored at -80 °C until further analysis. Assays were performed in technical duplicates.

### Assay analysis and calculation of kinetic HDR parameters

For quantification of HMBDP and total amounts of DMADP and IDP, LC-MS analysis was performed according to (González-Cabanelas et al. 2016). Initial velocities were calculated by plotting combined DMADP and IDP product concentrations over the different time points of the assays using OriginPro (2019) followed by a fit using a BoxLucasI model (Equation: y = a · (1–exp(−b · x)) on the data set. Initial velocity was calculated by fitting a linear regression through y(0,0)=a*b. Data were transformed into Lineweaver-Burk and Michaelis-Menten plots to determine *K*_m_ and *k*_cat_. DMADP:IDP ratios were determined by LC/MS-MS analysis using an Astec® Cyclobond® I 2000 column as previously described (Krause et al. 2020).

### Vector construction and transformation of poplar

For constructs overexpressing *PcHDR*2, the complete open reading frame was cloned into pCAMGW upstream of the maize (*Zea mays*) ubiquinone promoter (*ubi1*) promoter using Gateway Technology. The plasmid pCAMGW is a gateway-compatible version of pCAMBIA2301 (www.cambia.org). The protocol is published in detail by (Schmidt et al. 2010).

To knock-down the expression of *PcHDR*2, transgenic saplings were made carrying a *PcHDR*2 RNAi construct. For that a 164-bp region between position 57 and 221 of the coding sequence of *PcHDR*2 was selected, PCR-amplified, and cloned in sense and antisense orientations into the multiple cloning sites of the pTRAIN vector on either side of an intron as described by Levée et al. (2009). After HindIII digestion, the excised RNAi-cassette including also an upstream maize (*Zea mays*) ubiquitin promotor was ligated into the multiple cloning site of the pCAMBIA 1305.2 vector (www.cambia.org).

The *Agrobacterium tumefaciens*-mediated stable transformation of the *Populus* × *canescens* clone INRA 7171-B4 followed a protocol published by (Meilan and Ma 2006). Transgenic overexpressing as well as RNAi plants were amplified by micropropagation as described by (Behnke et al. 2007). Saplings of ∼10 cm high were repotted to soil (Klasmann potting substrate) and propagated in a controlled environment chamber for around six weeks (day, 22 °C; night, 18 °C; 65% relative humidity; 16 h/8 h light/dark cycle) before they were transferred to the greenhouse. All primers are listed in Supplemental Tabl. S3.

### Vector construction and transformation of spruce

To knock-down expression of *PaHDR*1 and *PaHDR*2, RNAi constructs were made employing a fragment between position 1,334 and 1,461 of the coding sequences of both *PaHDR*1 and *PaHDR2*. The same pCAMBIA 1305.2 vector used for the transformation of poplar was used for transformation of *Picea abies*. *Agrobacterium tumefaciens*-mediated stable transformation of *P. abies* embryogenic tissue (*Pa*186/3c) was performed as described in detail by (Schmidt et al. 2010). Generation of somatic transgenic seedlings was based on a protocol originally reported for white spruce from (Klimaszewska et al. 2005). Primers are listed in Supplemental Table S3.

### Isoprene and volatile terpenoid collection and analysis in *Populus* × *canescens*

Plants were enclosed with PET bags (“Bratschlauch”, Toppits, Minden, Germany) with the ends sealed (McCormick et al. 2014). Isoprene and terpenoid emission were collected using a push-pull-system attached to the PET bags. Charcoal filtered air was pumped into the bags with a flow rate of 1 L × min^−1^ and was pumped out with 0.8 L × min^−1^ while trapping the volatiles using different filters. After collection, leaf tissue was weighed to determine the total fresh weight (FW). Volatile collections were performed in individual measurements.

### Isoprene measurement

Isoprene emission was measured by a 10 min collection around noon. Compounds were trapped using fritted thermal desorption tubes filled with 60 mg Carbotrap® X, 20 to 40 mesh (Merck KGaA, Darmstadt, Germany) and glass wool. Before measurement, tubes were desorbed at 200 °C under a constant stream of nitrogen. After collection, tubes were closed with PTFE end caps and analyzed on a thermal desorption (TD-20) unit coupled to a GCMS-QP2010 system (Shimadzu, Kyoto, Japan). Volatiles were desorbed at 250 °C, trapped at −17 °C and released at 230 °C onto an OPTIMA-5 column (30 m × 0.25 mm × 0.25 µm; Macherey-Nagel, Düren, Germany) for analysis. Compounds were injected in split mode with a ratio of 10, a pressure of 83 kPa, a total flow of 18 mL × min^−1^ and a column flow as well as purge flow of 1.5 mL × min^−1^. Helium served as carrier gas. The temperature gradient was 40 °C for 3 min, increased to 50 °C by 0.5 °C × min^−1^, increased to 55 °C by 1.5 °C × min^−1^, increased to 300 °C with 70 °C × min^−1^ and held at 300 °C for 5 min. The GC was operated in scanning mode detecting masses with *m*/*z* 33 to 350. Isoprene was identified by using an authentic standard and comparison to reference spectra of the National Institute of Standards and Technology library.

### Volatile terpenoids

Volatiles were collected over 24 h to avoid day-to-night variation in emission rates, trapped using glass tubes filled with 20 mg Porapak-Q™ (http://www.volatilecollectiontrap.com) and eluted with 200 µL dichloromethane including 10 ng × µL^−1^ nonyl acetate as an internal standard. Qualitative and quantitative analysis of the samples was conducted using a 6,890 Series gas chromatograph (GC, Agilent Technologies) coupled to an Agilent 5,973 quadrupole mass selective detector (MS, interface temp, 270 °C; quadrupole temp, 150 °C; source temp, 230 °C; electron energy, 70 eV) or a flame ionization detector (FID) operated at 300 °C. Volatiles were separated using a ZB5 column (Phenomenex, Aschaffenburg, Germany, 30 m × 0.25 mm × 0.25 μm) and He (MS) or H_2_ (FID) as carrier gas. The sample (1 μL) was injected in splitless mode at an initial oven temperature of 45°C. The temperature was held for 2 min, then increased to 180°C with a gradient of 6 °C × min^−1^, and further increased to 300 °C with a gradient of 100 °C × min^−1^ and then held for 2 min. Compounds were identified by comparing their retention times and mass spectra to those of authentic standards and reference spectra in the Wiley and National Institute of Standards and Technology library.

### Volatile terpenoid, diterpenoid, carotenoid and chlorophyll extraction and analysis

Frozen tissue was extracted with 1 mL tert-butyl-methyl ether (TBME) for 20 h at room temperature including 50 µg × mL^−1^ 1,9-decadiene (Merck) and 47.3 µg × mL^−1^ dichlorodehydroabietic acid (CanSyn Chem Group, Toronto, Canada) for 20 h at room temperature. Vials were centrifuged, the ether phase made alkaline by adding 0.4 mL of 0.1 M (NH_4_)_2_CO_3_ (pH 8.0) solution and dehydrated using Na_2_SO_4_. Extracts were analyzed as described for poplar volatiles above.

For diterpenoid analysis in spruce, 200 µL of the ether phase was mixed with 25 µL 0.2 M N-trimethylsulfoniumhydroxide (TMSH; Macherey-Nagel) solution and incubated at room temperature for 2 h. Samples were analyzed using GC-FID and GC-MS measurements according to (Schmidt et al. 2011). The temperature gradient started at 150 °C for 3 min, increased to 280 °C with a gradient of 3.5 °C × min^−1^, and was held for 4 min. Compounds were identified by comparing their retention times and mass spectra to those of reference spectra in the Wiley and National Institute of Standards and Technology library.

Carotenoid and chlorophyll analysis was performed according to (Nagel et al. 2014).

### HMBDP, MEcDP, DMADP, IDP and short-chain prenyl diphosphate analysis

MEP pathway metabolites were extracted and analyzed as described in (Krause et al. 2020). Multiple reaction monitoring (MRM) was used to monitor parent ion to product ion formation. For DMADP/IDP and DMASP, parameters were used as described in (Krause et al. 2020). For MEcDP and HMBDP, parameters were as follows: HMBDP: *m*/*z* (Q_1_): 261; *m*/*z* (Q_3_): 79; declustering potential (DP): −60.0 V; collision energy (Ce): −36.0 V. MEcDP: *m*/*z* (Q_1_): 277; *m*/*z* (Q_3_): 79; DP: −50.0 V; Ce: −40.0 V. GDP, FDP and GGDP were analyzed according to (Nagel et al. 2014).

### Analysis of hemiterpene glycosides and measurement of DXS activity

Analysis was carried out to quantify the amounts of 2-*C*-methyl-D-erythritol (ME), ME-glycosides (ME- Glc), 4-hydroxy-3-methylbut-2-enol (HMB) and HMB-glycosides (HMB-Glc) according to (González-Cabanelas et al. 2015) and (Ward et al. 2012). The following mass transitions and parameters were used for MRM: ME: *m*/*z* (Q_1_): 135; *m*/*z* (Q_3_): 103; DP: −60.0 V; Ce: −10.0 V. ME-Glc: *m*/*z* (Q_1_): 297; *m*/*z* (Q_3_): 59; DP: −120.0 V; Ce: −10.0 V. HMB: *m*/*z* (Q_1_): 101; *m*/*z* (Q_3_): 83; DP: −55.0 V; Ce: −10.0 V. HMB-Glc: *m*/*z* (Q_1_): 263; *m*/*z* (Q_3_): 59; DP: −120.0 V; Ce: −10.0 V. The site of glycosylation in ME-Glc and HMB-Glc was not determined, making the MRM a sum parameter for all possible isomers. Data analysis was performed using Analyst Software 1.6.3 Build 1,569 (AB Sciex Instruments). Crude enzyme extract preparation and DXS assays were performed according to (Wright and Phillips 2014).

### Defense hormone analysis

The abundance of the phytohormones abscisic acid, jasmonic acid, (+) and (−) jasmonic acid-isoleucine, 12-oxophytodienoic acid, and hydroxylated forms of jasmonic acid was analyzed by LCMS-MS measurements as described in (Ullah et al. 2022).

### Determination of MEP pathway flux

MEP pathway flux was determined by following the incorporation of ^13^C label from ^13^CO_2_ into isoprene as described (Perreca et al. 2020). Briefly, plastidial concentrations of DXP, MEcDP, HMBDP and IDP + DMADP (combined pool) were estimated from MS measurements by comparing their final labeling fractions to that of isoprene, which was assumed to originate solely from plastidial sources. Isoprene labeling was followed on-line instantaneously with proton transfer reaction-mass spectrometry (PTR-MS). Following (Perreca et al. 2020), the flux was estimated by fitting the isoprene labeling time-course data to the following equation (see also Supplemental Fig. S9):


$$\begin{array}{c} \;f(t)=m\{1-\frac{A^{3}}{\left(A-B)(A-C)(A-D\right)}\exp\;\left(-\frac{J}{A}t\right)\\ -\frac{B^{3}}{\left(B-A)(B-C)(B-D\right)}\exp\;\left(-\frac{J}{B}t\right)\\ -\frac{C^{3}}{\left(C-A)(C-B)(C-D\right)}\exp\;\left(-\frac{J}{C}t\right)\\ \;-\frac{D^{3}}{\left(D-A)(D-B)(D-C\right)}\exp\;\left(-\frac{J}{D}t\right)\} \end{array}$$

where *f(t)* is the fractional labeling of isoprene as a function of time, *m* is the maximal fractional labeling at the end of the run, *A, B, C* and *D* are the plastidial pool sizes of DXP, MEcDP, HMBDP and IDP + DMADP, respectively, *J* is the pathway flux (fitted) and *t* is time. This equation is similar to equation (1) of (Perreca et al. 2020) but includes an additional term for HMBDP, which was measured here but not quantified in that analysis.

### Reverse transcription quantitative PCR (Rt-qPCR) and RNA-Seq analysis

For gene expression analysis, *Populus* × *canescens* and *Picea abies* cDNA was prepared as described above and appropriate primers were designed (Supplemental Table S3). Primer specificity was confirmed by melting curve analysis and sequencing of cloned amplicons. Species-specific primers for ubiquitin were used as a reference for the relative quantification of expression. The following PCR protocol was used: Initial denaturation at 95 °C for 3 min followed by 40 cycles of amplification (95 °C for 10 s and 60 °C for 20 s). Plate reads were recorded at the end of each cycle. Melting curve analysis was recorded after denaturing the samples at 95 °C for 2 min, measuring from 65 °C to 95 °C in steps of 0.5 °C. Analysis was performed on a Bio-Rad CFX Connect Real-Time PCR Detection system (Bio-Rad) in an optical 96-well plate, using SsoAdvanced™ Universal SYBR® Green Supermix (Bio-Rad). The baseline threshold was set to 200. All biological replicates were measured in technical triplicates.

Transcriptomes from herbivory-treated poplar leaves and infected poplar roots that had been previously obtained by (Guenther et al. 2019) and (Lackus et al. 2021) were screened for *HDR*1 and *HDR*2 expression. RPKM values of four biological replicates were obtained to compare control with herbivore and pathogen-treated plants.

### Statistical analysis

Statistical analysis was performed by comparing transgenic lines with empty vector controls using Student's t-test. Significance is shown by asterisks, representing *P*-values of ≤ 0.05, ≤ 0.01 and ≤ 0.001 with *, ** and ***, respectively.

## Supplementary Material

kiad110_Supplementary_Data

## Data Availability

The author responsible for distribution of materials integral to the findings presented in this article in accordance with the policy described in the Instructions for Authors (https://academic.oup.com/plphys/pages/General-Instructions) is: Axel Schmidt (aschmidt@ice.mpg.de).

## Supplemental data

The following materials are available in the online version of this article.

**Supplemental Figure S1**. HDR sequences from poplar, spruce, and other species.

**Supplemental Figure S2**. Phylogenetic analysis of HDR from poplar, spruce, and other species.

**Supplemental Figure S3**. *HDR* transcript abundance in different organs of *Populus trichocarpa* and *Picea abies* after herbivore, pathogen, or jasmonic acid/methyl jasmonate treatment.

**Supplemental Figure S4**. Effects of pH and temperature on the activity of recombinant HDR isoforms from *Populus* × *canescens* and *Picea abies*.

**Supplemental Figure S5**. Determination of initial velocities and *K*_m_ of recombinant HDR isoforms of *Populus* × *canescens* and *Picea abies* heterologously expressed in *E. coli*.

**Supplemental Figure S6**. Metabolic characterization and expression analysis of *PcHDR*2-silenced *Populus* × *canescens* lines with a lower silencing efficiency (∼10% of control transcript levels).

**Supplemental Figure S7**. *PaIDI* expression is not altered in transgenic *Picea abies* saplings silenced in *PaHDR*.

**Supplemental Figure S8**. Terpenoid intermediates and end products in transgenic *Picea abies* saplings silenced in *PaHDR* expression.

**Supplemental Figure S9**. Determining MEP pathway flux from fitting time-courses of ^13^C label incorporation into isoprene.

**Supplemental Table S1**. Effects of *PcHDR*2 gene silencing on the flux through the methylerythritol phosphate (MEP) pathway in transgenic *Populus × canescens* lines.

**Supplemental Table S2**. Quantification of phytohormone contents from transgenic *Populus* × *canescens* plants silenced in *PcHDR*2 expression.

**Supplemental Table S3**. Primer sequences and usage.
