# Supplementary material for: HDR, the last enzyme in the MEP pathway, differently regulates isoprenoid biosynthesis in two woody plants
Source: Plant Physiol. 2023 Feb 22;192(2):767–88. doi: 10.1093/plphys/kiad110 (PMC10231375; doi:10.1093/plphys/kiad110)
Supplement: kiad110_Supplementary_Data [file kiad110_supplementary_data.pdf]

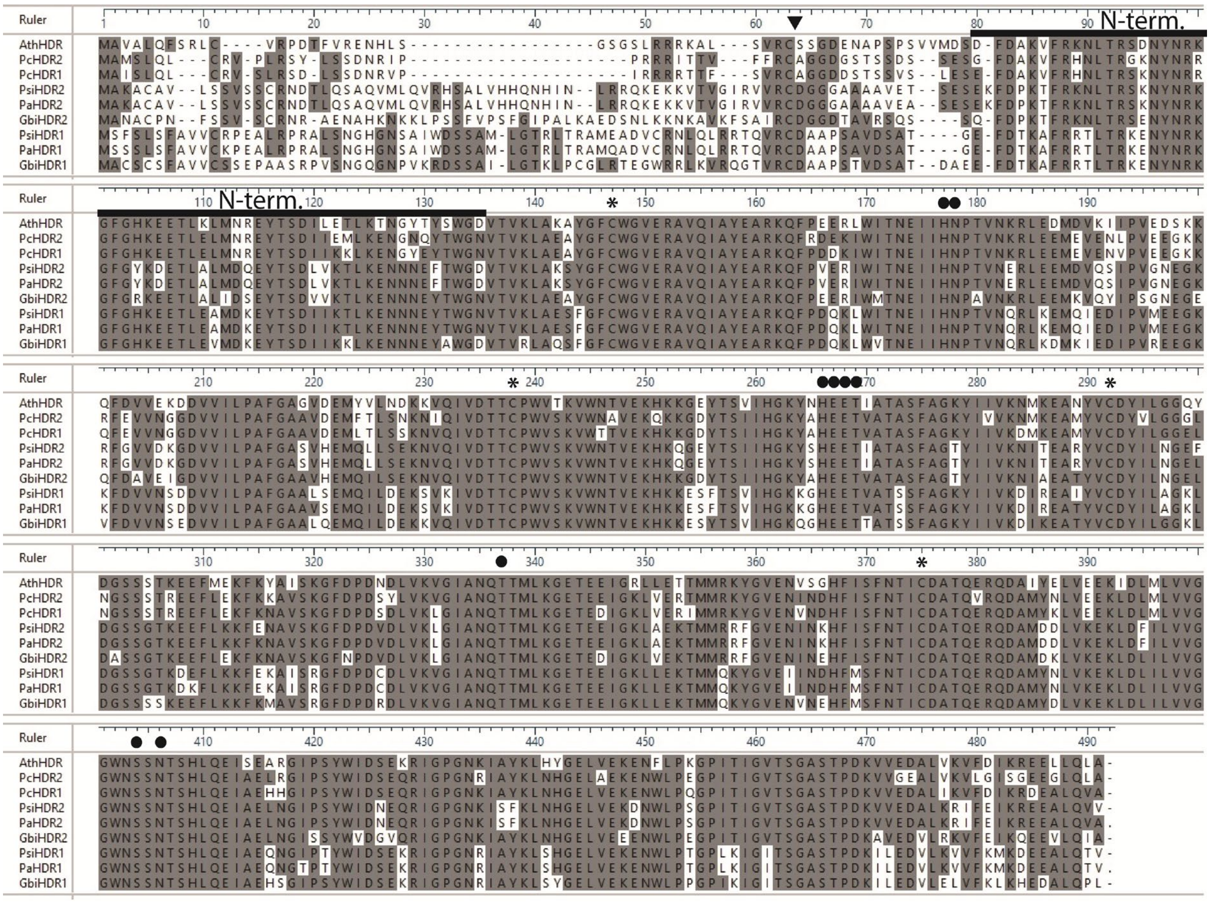

Supplemental Figure S1. HDR sequences from poplar, spruce, and other species. Amino acid sequences from different organisms were obtained from NCBI and congenie.org databases and aligned with MegAlign Pro ClustalW algorithm. Sequences with their Gene Bank accession numbers are *Ath*HDR (*Arabidopsis thaliana* HDR; AAW82381.1), *Pc*HDR2 (*Populus* × *canescens* HDR2; XP_002305413.1), *Pc*HDR1 (*Populus* × *canescens* HDR1; XP_002313816.1), *Psi*HDR2 (*Picea sitchensis* HDR2; ACN39959.1), *Pa*HDR2 (*Picea abies* HDR2; MA_105092g0010), *Gbi*HDR2 (*Ginkgo biloba* HDR2; ABB78089.1), *Psi*HDR1 (*Picea sitchensis* HDR1; ACN40284.1), *Pa*HDR1 (*Picea abies* HDR1; BT115538.1), *Gbi*HDR1 (*Ginkgo biloba* HDR1; ABB78088.1). Asterisks: four highly conserved cysteine residues; arrow: end of transit peptide; amino acids 80-130: N-terminal domain; black dots: critical amino acids in substrate binding site.

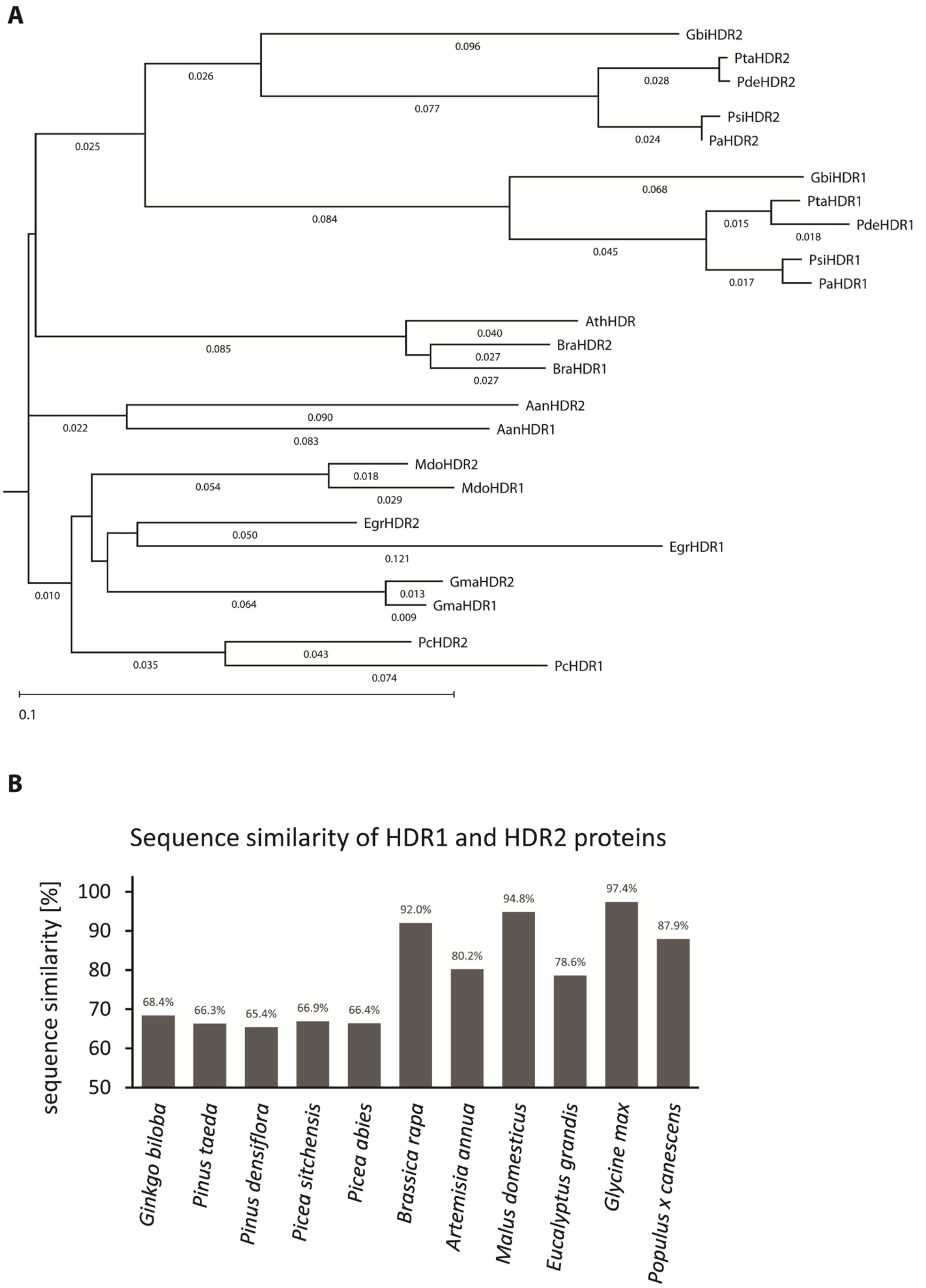

Supplemental Figure S2. Phylogenetic analysis of HDR from poplar, spruce, and other species. Amino acid sequences from different organisms were obtained from NCBI and

congenie.org databases and a phylogenetic tree was constructed using the MegAlign Pro ClustalW algorithm. Sequences with their Gene Bank accession numbers are *Aan*HDR1 (*Artemisia annua* HDR1; KY288069), *Aan*HDR2 (*Artemisia annua* HDR2; KX058541), *Ath*HDR (*Arabidopsis thaliana* HDR; AAW82381.1), *Bra*HDR1 (*Brassica rapa* HDR1; A00385), *Bra*HDR2 (*Brassica rapa* HDR2; H01210), *Egr*HDR1 (*Eucalyptus grandis* HDR1; I01241), *Egr*HDR2 (*Eucalyptus grandis* HDR2; C00644), *Gbi*HDR1 (*Ginkgo biloba* HDR1; ABB78088.1), *Gbi*HDR2 (*Ginkgo biloba* HDR2; ABB78089.1), *Gma*HDR1 (*Glycine max* HDR1; 12G046000), *Gma*HDR2 (*Glycine max* HDR2; 11G120900), *Mdo*HDR1 (*Malus domesticus* HDR1; MD10G1082800), *Mdo*HDR2 (*Malus domesticus* HDR2; MD05G1071900), *Pa*HDR1 (*Picea abies* HDR1; BT115538.1), *Pa*HDR2 (*Picea abies* HDR2; MA_105092g0010), *Pc*HDR1 (*Populus × canescens* HDR1; XP_002313816.1), *Pc*HDR2 (*Populus × canescens* HDR2; XP_002305413.1), *Pde*HDR1 (*Pinus densiflora* HDR1; EU439296.1), *Pde*HDR2 (*Pinus densiflora* HDR2; EU439297), *Psi*HDR1 (*Picea sitchensis* HDR1; ACN40284.1), *Psi*HDR2 (*Picea sitchensis* HDR2; ACN39959.1), *Pta*HDR1 (*Pinus taeda* HDR1; EF095154.1), *Pta*HDR2 (*Pinus taeda* HDR2; EF095155) (A). Sequences of the HDR isoforms from each species were aligned and sequence similarity was plotted. HDR1 and HDR2 from conifers differ much more between themselves than the isoforms from the other species (B).

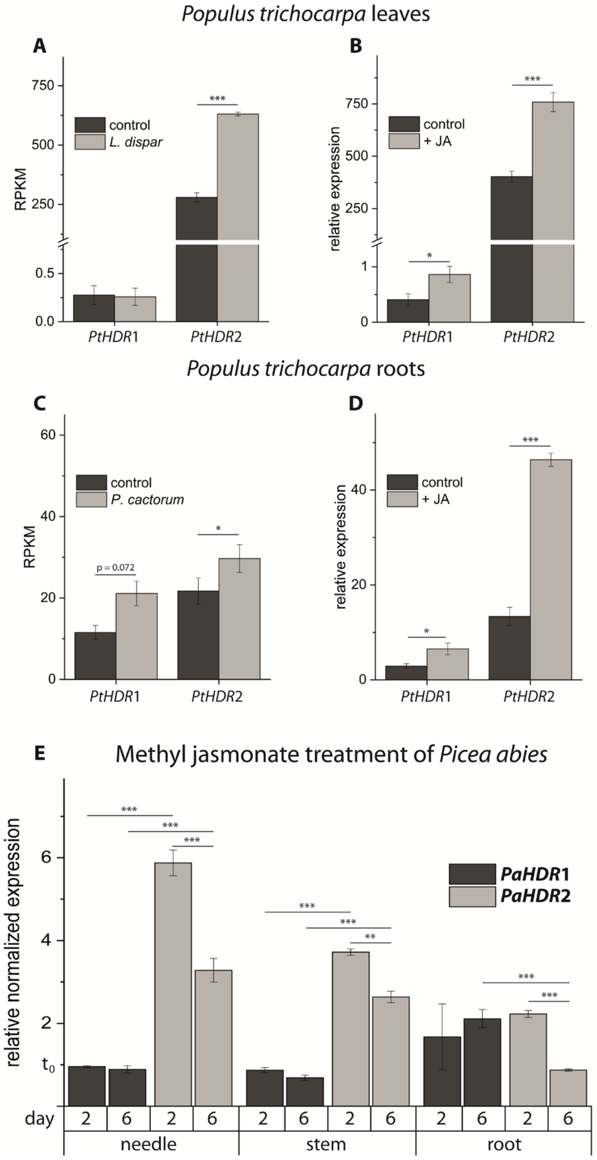

Supplemental Figure S3. *HDR* transcript abundance in different organs of *Populus trichocarpa* and *Picea abies* after herbivore, pathogen or jasmonic acid/methyl jasmonate treatment. *Populus trichocarpa* leaves were exposed to *Lymantria dispar* feeding and compared to control leaves without herbivory and RPKM (reads per kilobase of transcript per million mapped reads) values are presented. Data were extracted from transcriptomes published by Günther et al. (2019) (A). *Populus trichocarpa* leaves were analyzed for *PaHDR*1 and *PaHDR*2 expression by RT-qPCR after saplings were irrigated with 250 µmol ± jasmonic acid (B). Root transcriptomes were sequenced after infection by *Phytophtora cactorum* and RPKM values of *PcHDR* gene expression are presented. Data were extracted from transcriptomes published by Lackus et al. (2021) (C). Also *Populus trichocarpa* roots were treated with 250 µmol ± jasmonic acid as well and analyzed for *PaHDR*1 and *PaHDR*2 expression by RT-qPCR (D). Norway spruce organs were analyzed for *PaHDR*1 and *PaHDR*2 expression by RT-qPCR after saplings being sprayed with methyl jasmonate (MJ). Data were normalized to control organs before MJ treatment (t_0). Expression levels were measured two and six days after spraying. Values are given as mean ± standard deviation of four biological replicates (E). Statistical analysis was performed by using Student’s t-test.*** = $p < 0.001$; ** = $p < 0.01$; * = $p < 0.05$.

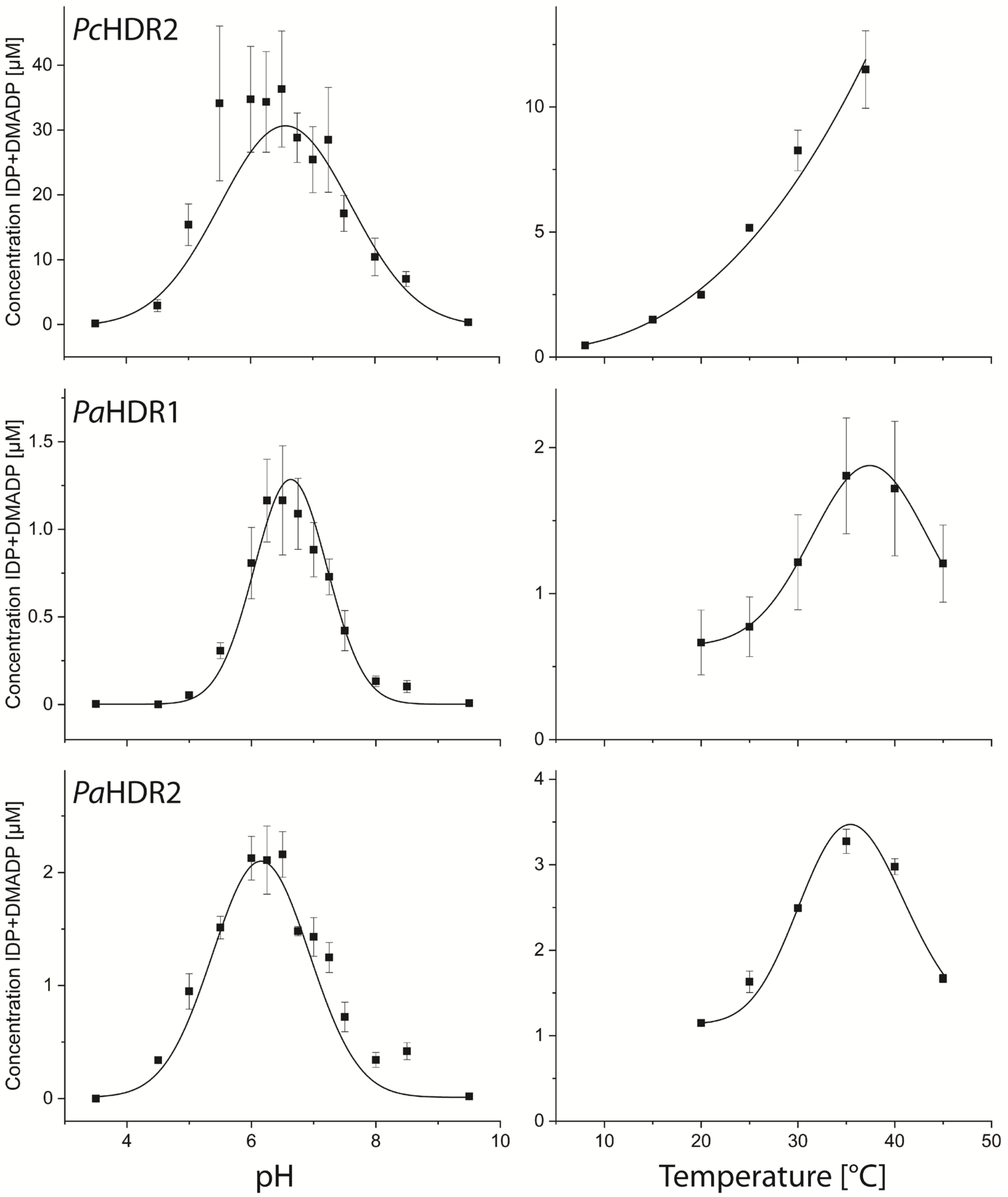

Supplemental Figure S4. Effects of pH and temperature on the activity of recombinant HDR isoforms from *Populus* × *canescens* and *Picea abies*. Proteins were heterologously expressed in *E. coli* and tested *in vitro* for their conversion rates of (*E*)-4-hydroxy-3-methylbut-2-en-1-yl diphosphate (HMBDP) to the combined products isopentenyl diphosphate (IDP) and dimethylallyl diphosphate (DMADP). Products were quantified by LC-MS/MS. All HDRs showed maximum activity at a pH between 6 and 6.5. Spruce *Pa*HDR enzymes had a temperature optimum at 35°C, while poplar *Pc*HDR2 did not show a peak value under the tested conditions. Each data point represents the mean ± standard deviation of two technical replicates each from two separate enzyme preparations.

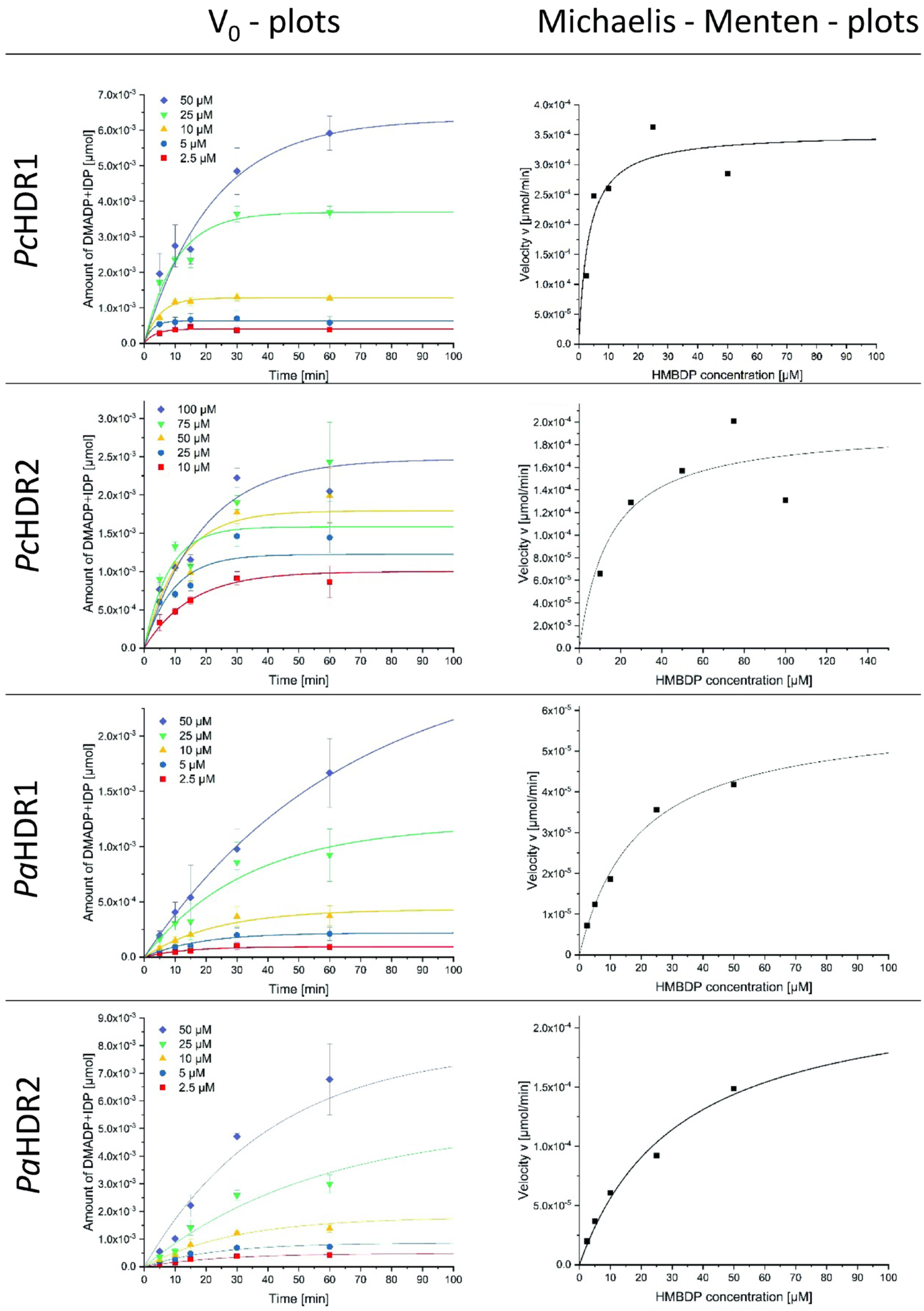

Supplemental Figure S5. Determination of initial velocities and K_m of recombinant HDR isoforms of *Populus* × *canescens* and *Picea abies* heterologously expressed in *E. coli*. Recombinant HDR isoforms were heterologously expressed in *E. coli* and tested for their conversion rates of (*E*)-4-hydroxy-3-methylbut-2-en-1-yl diphosphate (HMBDP) to the combined product pools of isopentenyl diphosphate (IDP) and dimethylallyl diphosphate (DMADP). Products were quantified by LC-MS/MS. Different substrate concentrations were applied and the reaction was allowed to run for 5, 10, 20, 30 and 60 min to obtain initial velocities for each concentration. Each data point represents the mean ± standard deviation of two technical replicates each from two separate enzyme preparations. Initial velocities were used to generate Michaelis-Menten-plots for the calculation of K_m and k_{cat}.

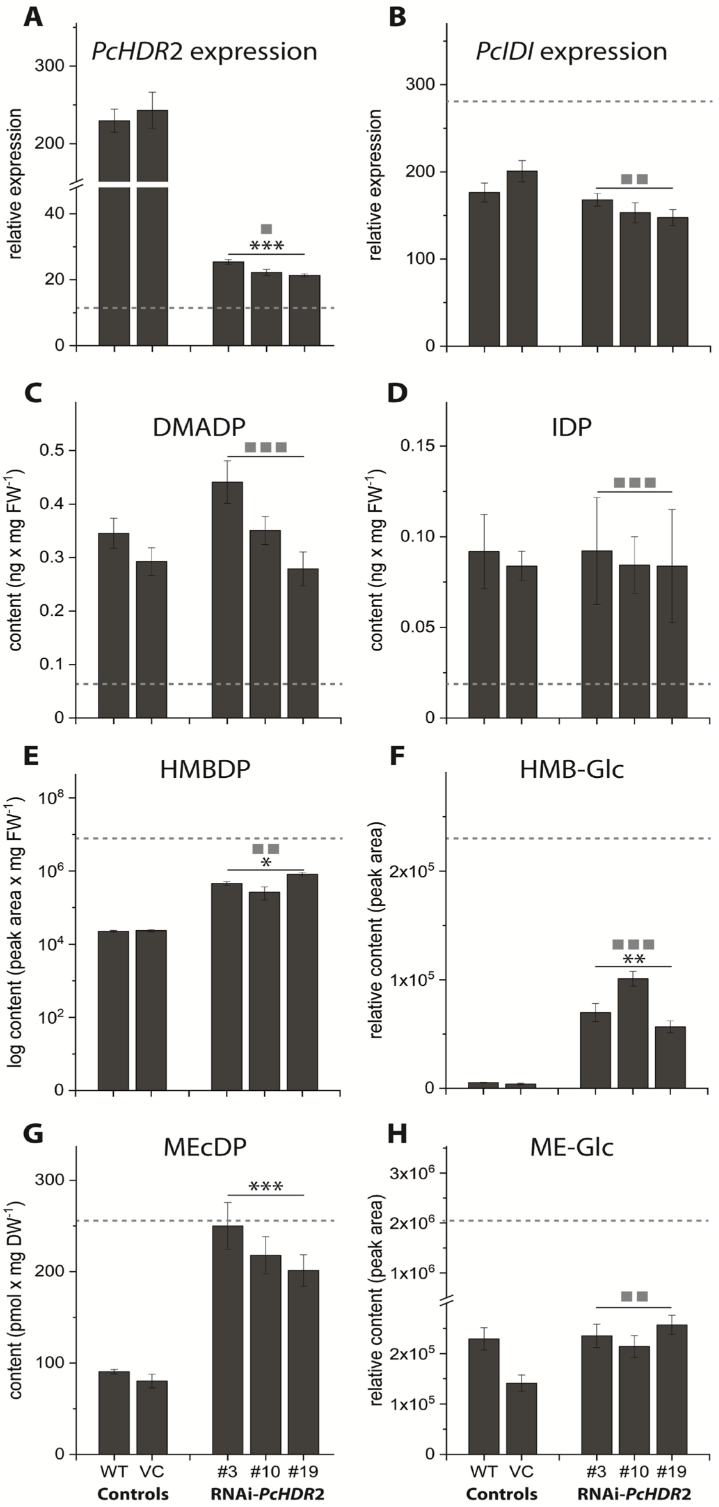

Supplemental Figure S6. Metabolic characterization and expression analysis of *PcHDR2*-silenced *Populus* × *canescens* lines with a lower silencing efficiency (~10% of control transcript levels). Transgenic lines presented in the main figures (see Figure 4) showed a silencing in *PcHDR2* expression that corresponds to 5% of the control lines. In this figure, we present data for plants with a lower silencing efficiency (only ~10% of control

transcript levels). The average values of expression and metabolite content in the 5% silenced lines are represented by gray dashed lines. Comparisons between 10% silenced land control lines are represented by black asterisks (∗), while comparisons between 10% silenced and 5% silenced lines are represented by gray squares (■) (A). *PcIDI* expression (B) and DMADP and IDP content (C and D) were not affected by lower silencing efficiency. Among MEP pathway intermediates, there was a significant accumulation of HMDP (E) and MEcDP (G), and the metabolites HMB-Glc (F), but not ME-Glc (H). Values are given as mean ± standard deviation of three biological replicates per line, with gray asterisks representing statistical comparison to the highly silenced lines and black asterisks representing the comparison to the controls. Statistical analysis was performed by using Student's t-test, ***/■■■ = $p < 0.001$; **/■■ = $p < 0.01$; */■ = $p < 0.05$; VC, vector control; HMBDP, (*E*)-4-hydroxy-3-methylbut-2-enyl diphosphate; MEcDP, 2-*C*-methyl-D-erythritol-2,4-cyclodiphosphate; HMB, (*E*)-4-hydroxy-3-methylbut-2-enol; ME, 2-*C*-methyl-D-erythritol.

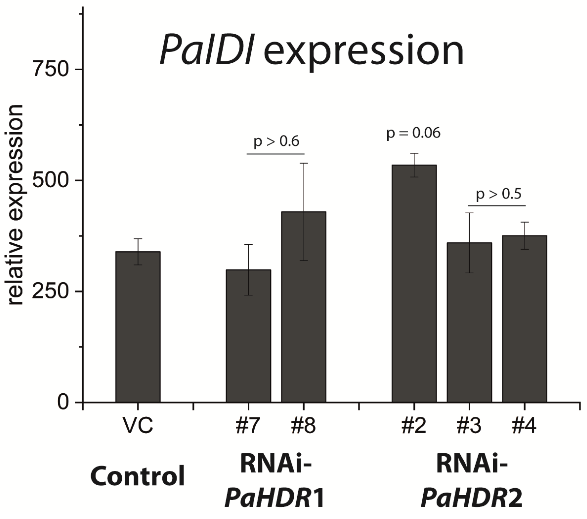

Supplemental Figure S7. *PaIDI* expression is not altered in transgenic *Picea abies* saplings silenced in *PaHDR* expression. RT-qPCR analysis was performed with two-year old spruce saplings. *PaIDI* expression was not affected by silencing of either *PaHDR*1 or *PaHDR*2. Values are given as mean ± standard deviation of at least four biological replicates per line. Statistical analysis was performed by using Student's t-test. No significant differences were measured between the control and the transgenic saplings.

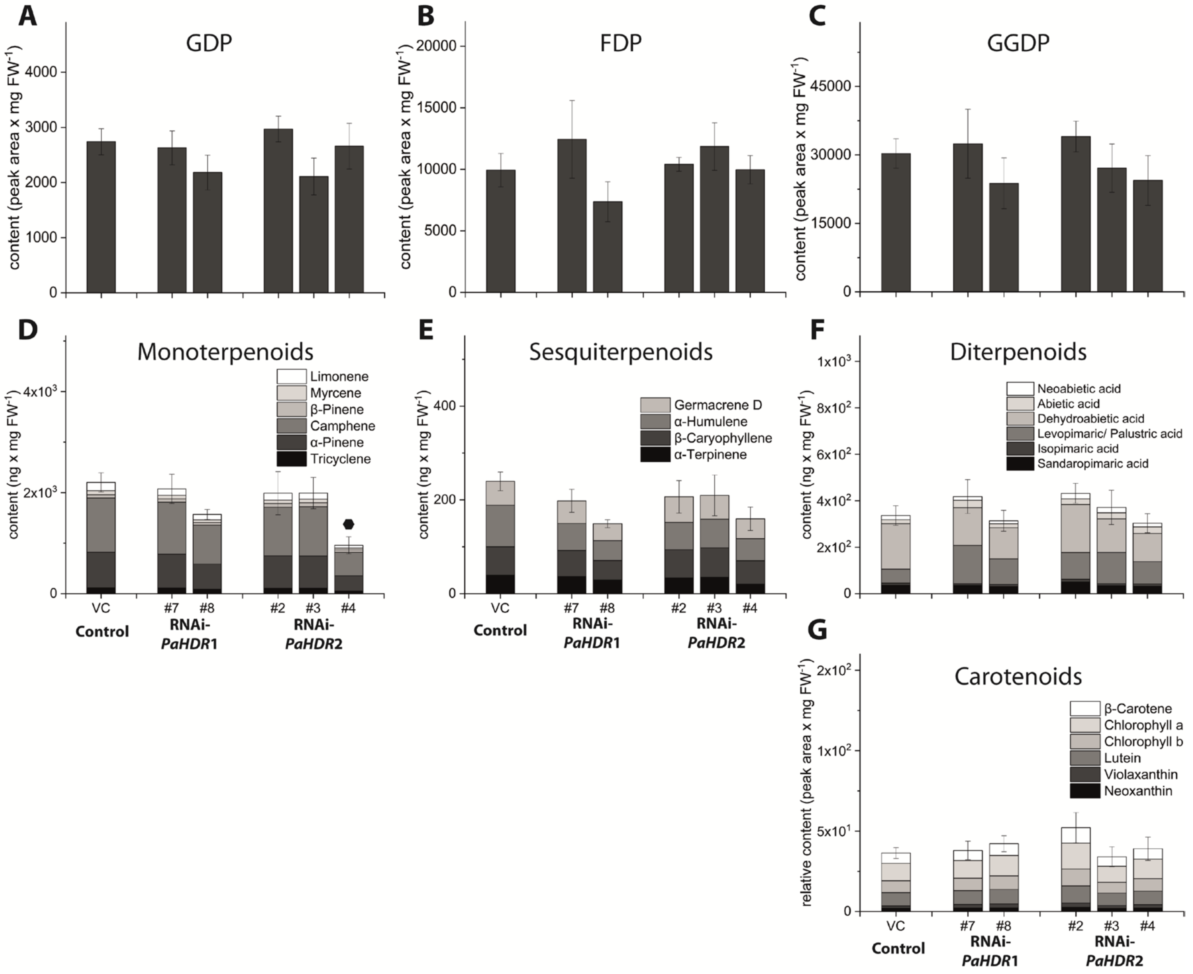

Supplemental Figure S8. Terpenoid intermediates and end products in transgenic *Picea abies* saplings silenced in *PaHDR* expression. Quantification of the prenyl diphosphates geranyl diphosphate (GDP; A), farnesyl diphosphate (FDP; B) and geranyl geranyl diphosphate (GGDP; C) showed no differences between transgenic and control lines. Total monoterpenoid content was reduced in only one *PaHDR*2-silenced line (⬢ = $p < 0.05$) (D). Sesquiterpenoids (E), diterpenoids (F) and carotenoids (G) were all not altered by *HDR* silencing. Values are given as mean ± standard deviation of at least four biological replicates per line, measured in technical triplicates. No significant differences were measured between the control and the transgenic saplings. Statistical analysis was performed by using Student's t-test, VC, vector control.

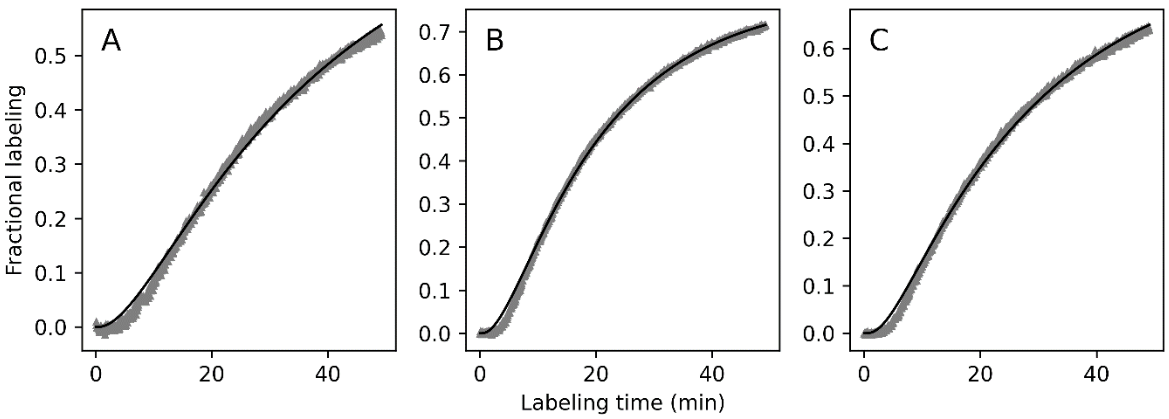

Supplemental Figure S9. Determining MEP pathway flux from fitting time-courses of ^{13}C label incorporation into isoprene. Shown are representative samples of fractional labeling of isoprene (grey symbols) as measured with PTR-MS for *PcHDR*2-silenced lines #2 (A), #7 (B) and #11 (C). Black lines indicate the fitted mathematical model used to calculate the flux (see main text, Materials and Methods).

Supplemental Table S1. Effects of *PcHDR2* gene silencing on the flux through the methylerythritol phosphate (MEP) pathway in transgenic *Populus* × *canescens* lines. Flux was determined from the incorporation of $^{13}CO_2$ into the emitted isoprene, analyzed on-line with a PTR-MS as described in the main text. Flux values are given ± standard error as estimated by the fitting algorithm (see main text). WT = wild type, VC = vector control.

**Line**	**Biological repeat**	**Flux (pmol.mg DW^{-1}.min^{-1})**
WT	1	36.20 ± 0.14
WT	2	18.93 ± 0.09
WT	3	26.50 ± 0.11
WT	4	28.70 ± 0.11
VC - #1	1	31.63 ± 0.07
VC - #1	2	24.42 ± 0.09
VC - #1	3	25.51 ± 0.09
VC - #1	4	27.74 ± 0.09
VC - #2	1	24.87 ± 0.11
VC - #2	2	39.82 ± 0.09
VC - #2	3	28.31 ± 0.11
VC - #2	4	32.49 ± 0.12
VC - #6	1	30.48 ± 0.08
VC - #6	2	29.70 ± 0.09
VC - #6	3	26.77 ± 0.07
VC - #6	4	26.40 ± 0.08
VC - #6	5	34.21 ± 0.11
RNAi-*PcHDR*2 - #2	1	42.13 ± 0.30
RNAi-*PcHDR*2 - #2	2	95.91 ± 0.68
RNAi-*PcHDR*2 - #2	3	45.64 ± 0.33
RNAi-*PcHDR*2 - #2	4	46.05 ± 0.33
RNAi-*PcHDR*2 - #2	5	31.74 ± 0.27
RNAi-*PcHDR*2 - #7	1	83.68 ± 0.49
RNAi-*PcHDR*2 - #7	2	32.87 ± 0.09
RNAi-*PcHDR*2 - #7	3	35.01 ± 0.21
RNAi-*PcHDR*2 - #7	4	17.23 ± 0.08
RNAi-*PcHDR*2 - #11	1	49.40 ± 0.22
RNAi-*PcHDR*2 - #11	2	19.12 ± 0.05
RNAi-*PcHDR*2 - #11	3	17.90 ± 0.09
RNAi-*PcHDR*2 - #11	4	25.43 ± 0.11

Supplemental Table S2. Quantification of phytohormone contents from transgenic *Populus* x *canescens* plants silenced in *PcHDR2* expression. Several plant hormones were quantified from transgenic and control plants. No significant differences were measured between the control and the transgenic saplings Values are given as mean ± standard deviation of four biological replicates per line, measured in technical triplicates. WT, wild type; VC, vector control.

Plant hormone content [ng/g FW]	WT			VC			RNAi-*PcHDR*2					
							#2			#11		
Jasmonic acid (JA)	7.2	±	2.3	11.3	±	2.0	9.6	±	0.9	12.9	±	5.2
Abscisic acid (ABA)	141	±	29	97	±	7	55	±	7	129	±	34
JA-Isoleucin (Ile)	0.56	±	0.08	0.64	±	0.05	0.66	±	0.19	1.13	±	0.67
12-oxo Phytodienoic acid (OPDA)	1543	±	682	1819	±	395	1875	±	304	1838	±	156
OH-JA	33.5	±	3.3	43.1	±	9.6	49.6	±	19.3	57.5	±	23.1
OH-JA-Ile	1.23	±	0.53	0.73	±	0.08	1.22	±	0.22	1.47	±	0.51
COOH-JA-Ile	0.20	±	0.11	0.11	±	0.01	0.34	±	0.05	0.70	±	0.34

Supplemental Table S3. Primer sequences and usage

No.	name	sequence	usage
1	*Pc*HDR1_qPCR_fwd	CGCCGTATAACCACCGTGT	RT-qPCR
2	*Pc*HDR1_qPCR_rev	TATGTCTGAACACTTTGGCGTC	RT-qPCR
3	*Pc*HDR2_qPCR_fwd	GGATGAGATGTTGACTTTGAGTAGC	RT-qPCR
4	*Pc*HDR2_qPCR_rev	AGGTATAATCTCCCTTCTTGTGCT	RT-qPCR
5	*Pc*IDI_qPCR_fwd	ACGTCAAGTACGTTAACCAGGA	RT-qPCR
6	*Pc*IDI_qPCR_rev	TGGTCCCACCACTTGAACAG	RT-qPCR
7	*Pc*IS_qPCR_fwd	ACACACAAACTGTTCAGAAATCCC	RT-qPCR
8	*Pc*IS_qPCR_rev	CCGTCTGGCTTCTGTTTCTGT	RT-qPCR
9	*Pc*DXS2_qPCR_fwd	AGGAACGAACAAGGTAGTCTCC	RT-qPCR
10	*Pc*DXS2_qPCR_rev	GTGGTATCGGCCCATCCAAG	RT-qPCR
11	*Pc*Ubi_qPCR_fwd	GTTGATTTTTGCTGGGAAGC	RT-qPCR
12	*Pc*Ubi_qPCR_rev	GATCTTGGCCTTCACGTTGT	RT-qPCR
13	*Pa*HDR1_qPCR_fwd	TGGACATGGGAATTCAGCCA	RT-qPCR
14	*Pa*HDR1_qPCR_rev	GCAGCATCGCACCTAACTTG	RT-qPCR
15	*Pa*HDR2_qPCR_fwd	AGGCCTCCGAGTCAGAGAAA	RT-qPCR
16	*Pa*HDR2_qPCR_rev	CATCAAGGCCAGCGTCTCAT	RT-qPCR
17	*Pa*IDI_qPCR_fwd	TGGAGGATACGACCATGGATG	RT-qPCR
18	*Pa*IDI_qPCR_rev	TCATGCCCAATGACATGATCTTC	RT-qPCR
19	*Pa*Ubi_qPCR_fwd	GTTGATTTTTGCTGGCAAGC	RT-qPCR
20	*Pa*Ubi_qPCR_rev	CACCTCTCAGACGAAGTAC	RT-qPCR
21	*Pc*HDR2_GW_pD15/17_for	GGGGACAAGTTTGTACAAAAAAGCAG GCTACTGCGCTGGCGGTGATGACTCTAC	cloning E. coli
22	*Pc*HDR2_GW_pD15/17_rev	GGGGACCACTTTGTACAAGAAAGCTG GGTCTTAAGCTACTTGTAAAGCTTCGTC	cloning E. coli
23	*Pc*DHR1_GW_pD15/17_for	GGGGACAAGTTTGTACAAAAAAGCAGGC TACTGCGCCGGCGGTGATGGCTCTAC	cloning E. coli
24	*Pc*DHR1_GW_pD15/17_rev	GGGGACCACTTTGTACAAGAAAGCTG GGTCTCATGCTAGTTGCAAGCCTTCCTC	cloning E. coli
25	*Pa*HDR1_GW_pD15/17_for	GGGGACAAGTTTGTACAAAAAAGCAG GCTACTGCGATGCTGCTCCCAGCGCTGTAG	cloning E. coli

26	*Pa*HDRT_GW_pD15/17_rev	GGGGACCACTTTGTACAAGAAAGCTG GGTCTTATACTGTCTGCAACGCCTCCTC	cloning E. coli
27	*Pa*HDR2_GW_pD15/17_for	GGGGACAAGTTTGTACAAAAAAGCAG GCTACTGCGATGGAGGGGGAGCTGCTGCTG	cloning E. coli
28	*Pa*HDR2_GW_pD15/17_rev	GGGGACCACTTTGTACAAGAAAGCTG GGTCCTATGCTACTTGCAGAGCCTCTTC	cloning E. coli
29	PcHDR2 OE for	GGGGACAAGTTTGTACAAAAAAGCAGG CTTAATGGCTATCTCTCTCCAACTCTGC	cloning poplar
30	PcHDR2 OE rev	GGGGACCACTTTGTACAAGAAAGCTG GGTCTTAAGCTACTTGTAAAGCTTCGTC	cloning poplar
31	PcHDR2 RNAi for	TGCTCTAGAGCAACAATCGCGTACCTAT CCGC	cloning poplar
32	PcHDR2 RNAi rev	CGGGATCCCGTGTGGCCAAAACCTC TACGA	cloning poplar
33	PaHDR1 RNAi for	TGCTCTAGAGCAGGAAAATTGGCTTCC AACCG	cloning spruce
34	PaHDR1 RNAi rev	CGGGATCCCGGTCTGCAACGCCTC CTCATC	cloning spruce
35	PaHDR2 RNAi for	TGCTCTAGAGCAAGATAACTGGCTGCC ATCAGGC	cloning spruce
36	PaHDR2 RNAi rev	CGGGATCCCGACTTGCAGAGCCTCTTC TCGTTTG	cloning spruce
